# Bottom-Up Synthesis of Platinum Dual-Atom Catalysts on Cerium Oxide

**DOI:** 10.1021/acscatal.4c01840

**Published:** 2024-06-17

**Authors:** Martijn J. Mekkering, Petrus C. M. Laan, Alessandro Troglia, Roland Bliem, Ali C. Kizilkaya, Gadi Rothenberg, Ning Yan

**Affiliations:** † Van’t Hoff Institute for Molecular Sciences, University of Amsterdam, Science Park 904, 1098 XH Amsterdam, The Netherlands; ‡ Advanced Research Center for Nanolithography (ARCNL), Science Park 106, 1098 XG Amsterdam, The Netherlands; § Department of Chemical Engineering, Izmir Institute of Technology, 35430 Urla, Izmir, Turkey; ∥ Key Laboratory of Artificial Micro- and Nano-Structures of Ministry of Education, School of Physics and Technology, Wuhan University, Wuhan 430072, China

**Keywords:** pre-organization, metal−support interaction, hydrogen generation, double-atom catalysis, water activation

## Abstract

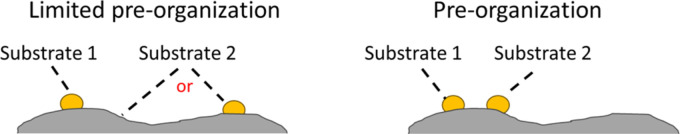

We present here the synthesis and performance of dual-atom catalysts (DACs), analogous to well-known single-atom catalysts (SACs). DACs feature sites containing pairs of metal atoms and can outperform SACs due to their additional binding possibilities. Yet quantifying the improved catalytic activity in terms of proximity effects remains difficult, as it requires both high-resolution kinetic data and an understanding of the reaction pathways. Here, we use an automated bubble counter setup for comparing the catalytic performance of ceria-supported platinum SACs and DACs in ammonia borane hydrolysis. The catalysts were synthesized by wet impregnation and characterized using SEM, HAADF-STEM, XRD, XPS, and CO-DRIFTS. High-precision kinetic studies of ammonia borane hydrolysis in the presence of SACs show two temperature-dependent regions, with a transition point at 43 °C. Conversely, the DACs show only one regime. We show that this is because DACs preorganize both ammonia borane and water at the dual-atom active site. The additional proximal Pt atom improves the reaction rate 3-fold and enables faster reactions at lower temperatures. We suggest that the DACs enable the activation of the water–O–H bond as well as increase the hydrogen spillover effect due to the adjacent Pt site. Interestingly, using ammonia borane hydrolysis as a benchmark reaction gives further insight into hydrogen spillover mechanisms, above what is known from the CO oxidation studies.

## Introduction

The chemistry of single-atom catalysts (SACs) has attracted much attention in the past decade. The main driving force is the increasing prices of scarce metals and the theoretical simplicity of the active sites.^1−6^ Yet the same boon can also hinder some chemical reactions due to the lack of potential binding sites. In theory, one could solve this problem by adding a second catalytically active atom, similar to the evolution of bimetallic enzymes such as urease and methane monooxygenase.^7,8^ Indeed, such dual-atom catalysts (DACs) have been a long-standing target in heterogeneous catalysis.^9−12^ In theory, DACs combine the advantages of 100% metal utilization with the possibility of using adjacent active sites.

There are few publications on the preparation and/or upscaling of DACs. Atomic layer deposition was used to synthesize high-quality SACs and DACs.^13^ Alternatively, one can use mono- or dinuclear precursor complexes,^14^ the decomposition of which yields isolated metal sites. Yet the synthesis of stable SACs and DACs remains a challenge.^15−18^

Here, we present a simple and general protocol for incorporating single- and dual-atom Pt sites onto a ceria support, thereby creating stable SACs and DACs. The method is based on wet impregnation of specific precursors, followed by drying and calcination under controlled conditions. The catalysts were characterized by scanning electron microscopy (SEM), high-angle annular dark field scanning electron microscopy (HAADF-STEM), X-ray diffraction (XRD), X-ray photoelectron spectroscopy (XPS), and carbon monoxide diffuse reflectance infrared Fourier transform spectroscopy (CO-DRIFTS) and tested for activity using ammonia borane hydrolysis (eq 1). Traditional studies on SACs focus on oxygenation reactions, especially the conversion of CO to CO_2_.^19−23^ However, we chose ammonia borane hydrolysis on ceria-supported catalysts because the background reaction is minimal, which makes understanding proximity effects easier (in contrast to CO oxidation studies^19,24,25^). The influence of the metal–support interaction was controlled by depositing platinum on the same facet. This reaction has more than just academic value, as ammonia borane can be used as a hydrogen storage medium thanks to its high hydrogen content (19.6% w/w).^26^ We chose a high-resolution bubble counter setup over an online GC, as the latter is limited by a time resolution of ca. 2 min because the only gaseous product is hydrogen.
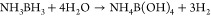
1

The reaction proceeds readily in the presence of noble metal atoms but gives only traces of the product in the absence of a catalyst. While the exact mechanism is still under debate, ammonia borane likely undergoes splitting of the B–N bond as oxygen from water attacks the boron following an S_N_2 pathway.^27^ The hydridic BH_3_ hydrogens react with the protic ones of H_2_O, giving molecular hydrogen.^28^ It is known that hydrogen can transfer over reducible metal oxides.^29^ Thus, we hypothesized that DACs could improve the hydrogen transfer efficiency because the protic and hydridic hydrogens would form in proximity and thus could more easily combine into molecular hydrogen. Our results show that DACs are much more active than SACs in this reaction, confirming the advantage of the dual-atom active sites.

## Results and Discussion

### Synthesis and Characterization of Platinum SACs and DACs

**Figure 1:**
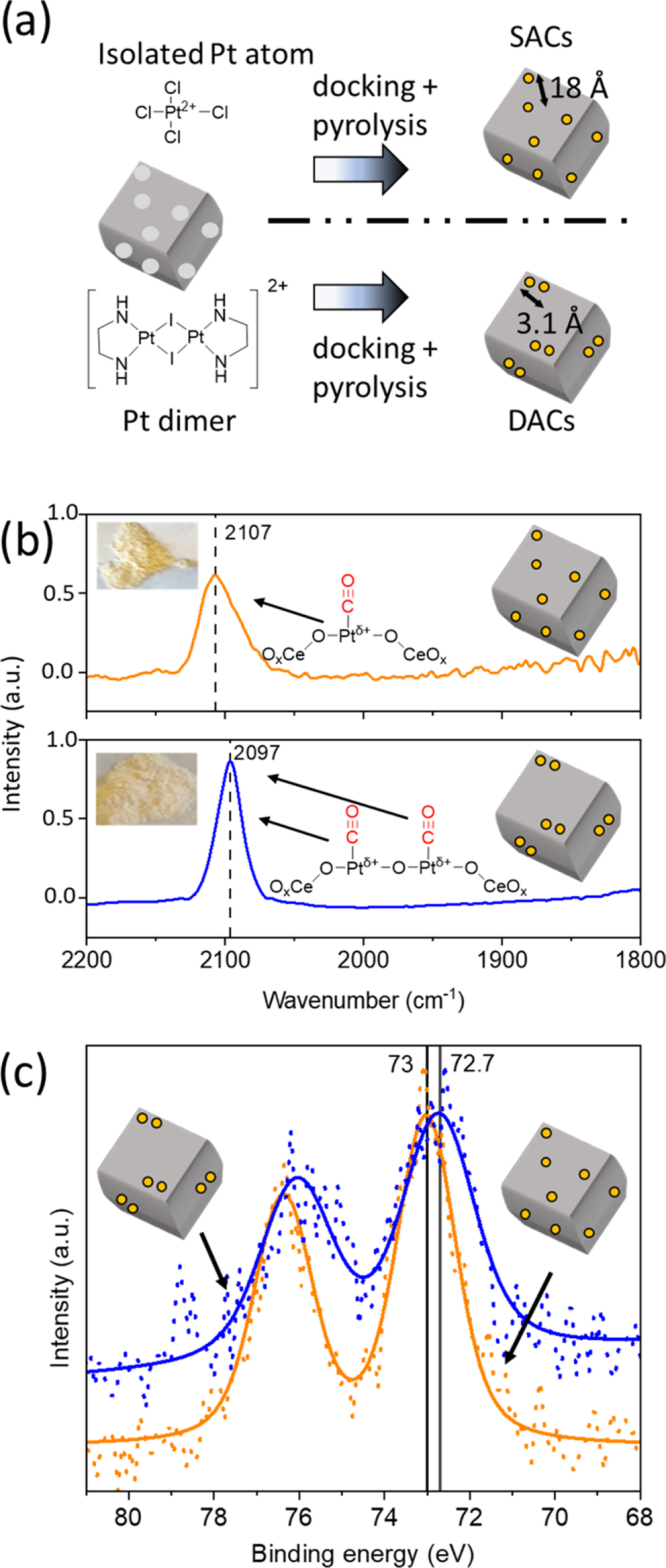
(a) Synthesis of SAC and DAC samples. The lighter parts on the cerium oxide support indicate defect sites. (b) CO-DRIFTS of the SAC (orange) and DAC (blue). The inset images are the measured powders. (c) XPS of the SAC (orange) and the DAC (blue), both with a one-component fitting with background. Note that the doublet in the XP spectrum of the DAC is broader than the SAC and the peak at 79 eV is noise.

We chose ceria as our support due to its known capacity for trapping platinum atoms^17,30−32^ and, therefore, prepared CeO_2_ cubes with preferentially exposed (100) facets. The morphology and crystal structure were confirmed by SEM and pXRD (Figures S1 and S2, respectively). We then prepared the platinum SAC and DAC samples (Figure 1a). The SAC and DAC sites were deposited on the support by wet impregnation. The SAC was prepared using a chloroplatinate hexahydrate precursor (H_2_Pt^II^Cl_6_)·6H_2_O, and the DAC was prepared using the dinuclear precursor^33^ [Pt_2_^II^I_2_(H_2_NCH_2_CH_2_NH_2_)_2_](NO_3_)_2_ (see Experimental Section for details). The platinum surface loading of both samples was similar, as confirmed with CO-DRIFTS and XPS (see Figures 1b,c and S6).

The interatomic distances of the SAC were based on the weight loading (0.08 wt %), the N_2_ physisorption BET surface area (8 m^2^/g, see calculation and Figure S5), and the molecular weight of platinum (197 g/mol). The drying and calcination steps are crucial here, as we needed to ensure the same metal–support interaction for both catalysts.^34^ Thermogravimetric analysis following the work of Schweizer et al.^35^ gave us the decomposition temperature of SAC salt (cf. also the TGA analysis of the DAC in Figure S3). We found that 500 °C was the optimal calcination temperature for both the SACs and the DACs, as both precursors decompose completely. Moreover, HAADF-STEM imaging showed no Pt clusters or larger particles, supporting the formation of single-atom and dual-atom sites (Figure S7).

The CO-DRIFTS measurements on the SAC and DAC samples showed the site-isolated atoms or pairs (see Figure 1b; the insets show photos of the samples). The SAC and DAC look the same by visual inspection. The linearly bonded carbonyl group is seen at 2107 cm^–1^.^25,36^ Interestingly, in the DAC, this peak is shown at 2097 cm^–1^. To the best of our knowledge, no CO-DRIFTS measurements have been reported for Pt DACs because other Pt DAC catalysts that were prepared are based on carbon materials, which complicate the IR analysis.^13^ We therefore hypothesize that this decrease in energy is likely caused by the difference in placement between the SACs and the DACs.^37^ The SAC sites will form at surface defects or high-energy sites. These sites bind strongly to the Pt atoms, reducing the chance of pi back-donation from the Pt d orbitals to the carbonyl p orbitals. This is supported by the much lower CO stretching frequency peak for clusters at lower frequencies.^38^ Additionally, no Pt clustering was observed, as there is no peak at ∼1820 cm^–1^.^39^ Control experiments confirmed that the pristine support itself is PGM-free (Figure S4).

To ascertain the experimentally assigned vibrational peaks for CO adsorbed on our SACs and DACs, we performed DFT modeling of a representative CeO_2_ surface doped with single and double Pt atoms, denoted as Pt_1_–CeO_2_(111) and Pt_2_–CeO_2_(111), respectively. The calculated Pt–Pt distance was 3.62 Å for the Pt_2_–CeO_2_(111) and 3.83 Å for CO–Pt_2_–CeO_2_(111) (see Table S1). The optimization of Pt-substituted CeO_2_(111) shows that Pt doping decreases the Ce–Ce distances around Pt while it increases the Ce–O distances. Furthermore, for the CO-adsorbed structures, Pt atom(s) move into the subsurface (see the side views in Figure S18), leading to further elongated Ce–O bond lengths. This indicates that doping of Pt atoms can facilitate the formation of oxygen vacancies, which was previously reported also for Ga doping of CeO_2_(111) surfaces.^40^

Related to the simulation of CO vibrational spectra on Pt_X_-CeO_2_(111), we ran optimization calculations that show that CO adsorbs linearly on both single and double Pt atoms (thus not in a bridge configuration; see Figure S18). The calculated CO stretching frequencies were 2099 cm^–1^ on Pt_1_–CeO_2_(111) and 2093 cm^–1^ on Pt_2_–CeO_2_(111). This decrease matches our experimental assignments of the CO stretching frequency on Pt_1_-and Pt_2_-doped CeO_2_ surfaces, indicating a small red shift in the CO vibrational spectra when moving from single to double Pt catalysts.

Figure 1c shows the Pt 4f range of the XP spectra of the SAC and DAC samples. Both show a similar peak shape and follow a single component fit of the Pt^2+^.^41^ Because the peaks are Gaussian, we suggest that no Pt^0^ clusters are formed. Moreover, the peak at 79 eV is considered noise, as the background intensity is relatively high. This is further supported by the minimal width of the peak (FWHM = 2 eV) compared to the other Pt peaks. Importantly, we do not see any Cl^–^ nor I^–^ residuals on the ceria surface either (Figure S8), precluding reaction interference from these halides as discussed below. Further, ICP analysis confirmed that the total platinum contents were 0.06 wt % for the DAC and 0.08 wt % for the SAC. Similar ratios were estimated from their respective CO–DRIFTS integrals (see fits in Figure S6) and the surface weight loading estimated with XPS, which was 4:5 for the DAC:SAC.

**Figure 2:**
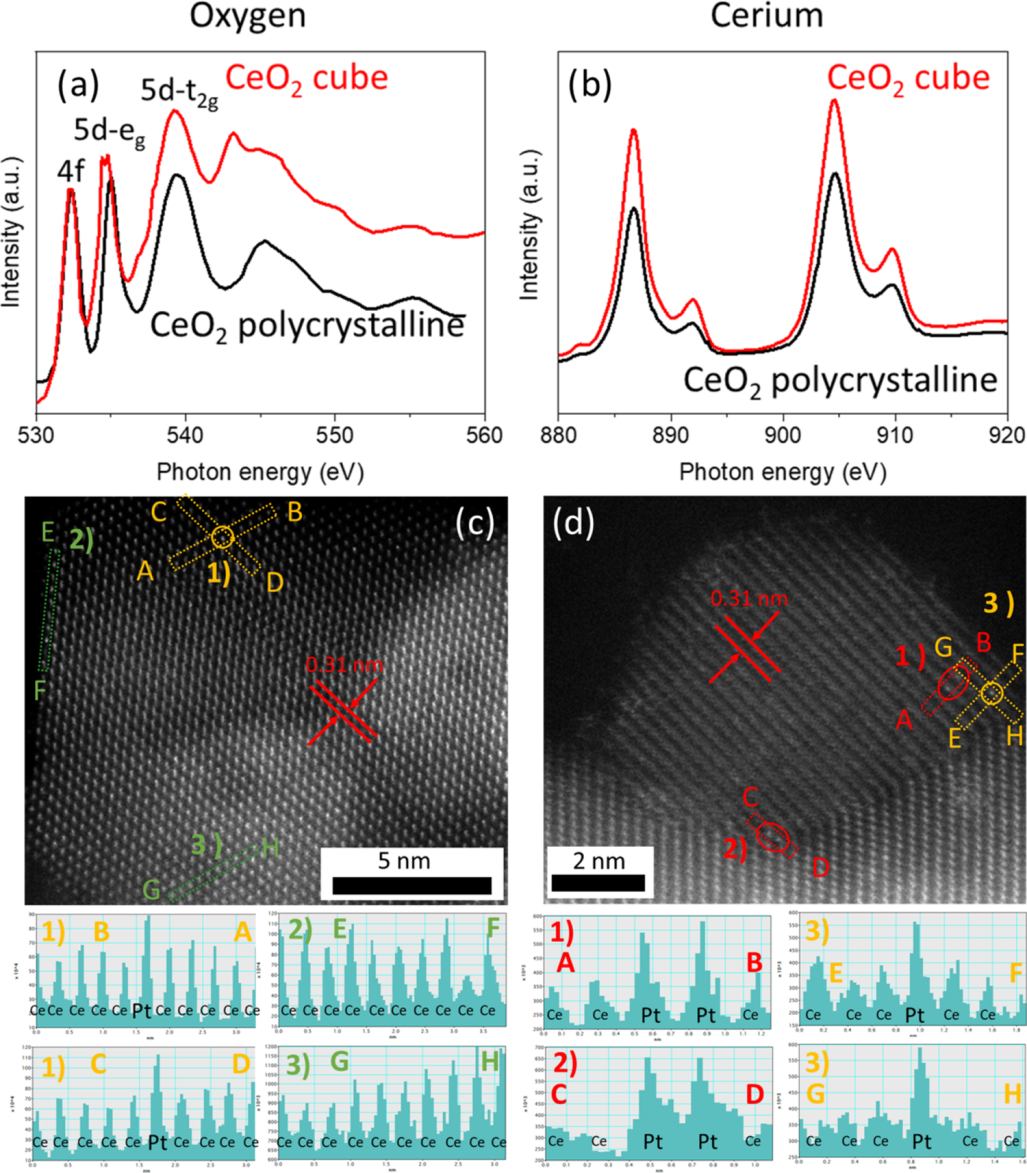
XAS measurements (a, b) of the oxygen K-edge and the cerium M_5,4_-edge of the cube with reference CeO_2_. AC-HR-TEM measurements of SAC (c) and DAC (d). The inserted circles are the indicated platinum atoms, and the rectangles indicate the line scans, as shown under the respective figures.

Figure 2a,b shows the X-ray near-edge structure spectroscopy (XANES) of the oxygen K-edge and the cerium M_5,4_-edge of the cubic cerium oxide and the reference sample with the identified peaks.^42^ The fittings of the cerium M_5,4_-edge peaks are shown in Figure S9. Dividing the peak integral at 886.6 eV by 904.6 eV results in 0.675 and 0.716 for the polyhedral and cubic CeO_2_, respectively.^43^ This indicates the presence of oxygen vacancy clusters. Together with the electron paramagnetic resonance measurements (EPR, Figure S10), this suggests that there are a lot of anchoring sites (the defect sites) for the platinum atoms or pairs on the CeO_2_ cubes.

Next, we measured aberration-corrected high-resolution transmission electron microscopy (AC-HR-TEM) of the SAC (Figure 2c) and DAC (Figure 2d) to confirm the atomic deposition of Pt. The circles and ovals indicate isolated Pt atoms and Pt pairs, respectively. We again confirmed the cubic lattice structure of CeO_2_, and an interplanar distance of 0.31 nm was measured, as shown in red.^44^ The intensity profiles of the CeO_2_ cubic lattice structure are shown in the figures below the HR-TEM image. The intensity differences can be due to (i) doping of the lattice with Pt, (ii) orientation differences of the CeO_2_ particle with the beam and/or detector, or (iii) thickness variations of the measured specimen across the surface plane. We performed additional line scans to exclude the above-mentioned causes, and these are shown in Figures S12–S14.

To eliminate the contribution of the latter reasons, we performed line scans on the edges of the cerium oxide, as indicated by the dashed rectangles in the HR-TEM images. Peaks in both the x and y direction indicate a site-isolated Pt atom (SACs–yellow, Figure 2c), and a double peak in one direction indicates an isolated pair of Pt atoms (DACs–red, Figure 2d). We also compared these with line scans on several regions where no bright dot was visually observed to further confirm the effectivity of the line scan (cf. the green rectangles in Figure 2c, where no peak is observed). Note that both the Pt atoms for both the SAC and the DAC are incorporated into the fluorite lattice structure of CeO_2_.^45^ Moreover, the calculated Pt–Pt distance is roughly 0.31 nm, being slightly smaller than the Pt–Pt distance of 0.38 nm in the Pt_2_ precursor^46^ (and matching with the interplanar distance of CeO_2_ as mentioned above). We also see a site-isolated atom in the DAC (Figure 2d, shown in yellow). This could (i) still have an adjacent Pt atom next to it but is unclear in the image, (ii) be an isolated atom due to partial decomposition of the Pt_2_ precursor during catalyst preparation, (iii) be caused by beam damage of a DAC or (iv) be a site-isolated species due to significant Pt migration on CeO_2_ at higher calcination temperatures and limited anchoring during preparation. We therefore presume that most platinum atoms on the surface of the DAC are in pairs with some isolated atoms (Figure S11 shows the AC-HR-TEM images without the rectangles).

### Catalytic Hydrolysis of Ammonia Borane with SACs and DACs

**Figure 3:**
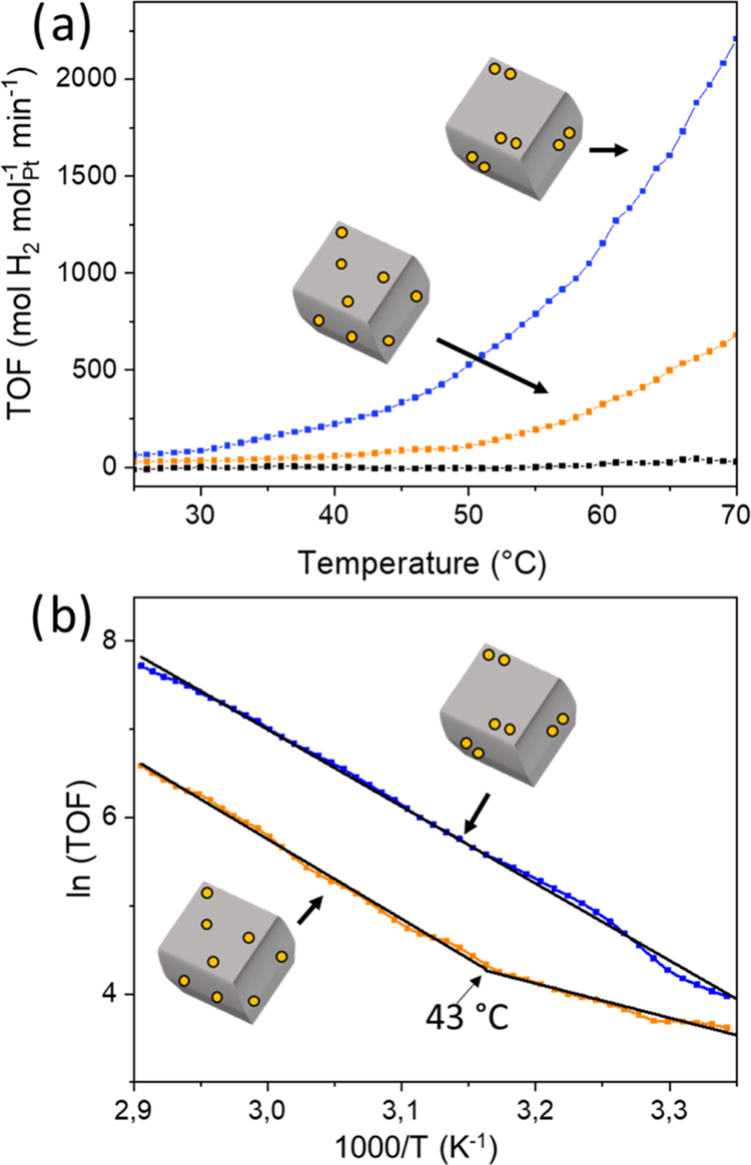
(a) Ammonia borane hydrolysis experiments at different temperatures for the support (black), the SAC (orange), and the DAC (blue). (b) Arrhenius plot of figure (a); the support was left out for clarity. These are high-precision measurements: Each point on the curve represents a window average of 25 data points, and each curve is an average of duplicate experiments.

Subsequently, we ran ammonia borane hydrolysis experiments with both catalysts between 25 and 70 °C, as well as blank experiments using only the ceria support (Figure 3a). The blank support (black curve, no platinum) shows minimal conversion in the studied temperature range. Comparing the turnover frequencies (TOF) showed a clear difference between the Pt-containing catalysts and the blank support (the TOF values of the SACs and the DACs are corrected for the samples’ Pt ratio). Importantly, we also see a large difference between the SAC and the DAC samples: a 3-fold improvement of the catalytic performance at each temperature when using the DAC. We hypothesize that this increase pertains to a difference in proximity effects; see below.

Figure 3b shows the corresponding Arrhenius plots for these reactions. We see a straight line for DAC with a calculated activation energy of 18.0 ± 0.8 kcal mol^–1^. Conversely, the SAC gives two different slopes, above and below 43 °C. The calculated activation energy above 43 °C is 17.3 ± 0.5 kcal mol^–1^, highly similar to that of the DAC (below 43 °C, there is practically no reaction taking place for the SAC). The fittings of these Arrhenius plots are given in Figure S16. Note that Figure 2b compares ln(TOF) rather than the more conventional ln(*k*), to correct for any difference in the samples’ Pt content. There is ongoing debate about whether the rate-determining step is O–H activation or B–H activation, but there is more compelling evidence for an O–H rate-determining step.^47−52^ Earlier work done on related systems shows that the activation energy increases by decreasing the weight loading, which also increases the activation energy. The authors concluded that the single-atom limit was not yet reached as the activation energy still increased.^53^ However, with our precise kinetic measurements, we can go one step further by assessing the influence of the dual-atom site on the rate-determining step. By using the high-resolution kinetics on atomically controlled catalysts, we suggest that the rate-determining step remains the same but the additional platinum atom does activate water partially. To exclude the effect of the different counterion (Cl^–^ or I^–^), we ran experiments with a 1:2 molar ratio of Pt for both the SAC and DAC to the corresponding ion (see Figure S17). Here, we observed a similar catalytic performance after the addition of the corresponding halide salts and concluded that the counterion did not influence the reaction.

**Figure 4:**
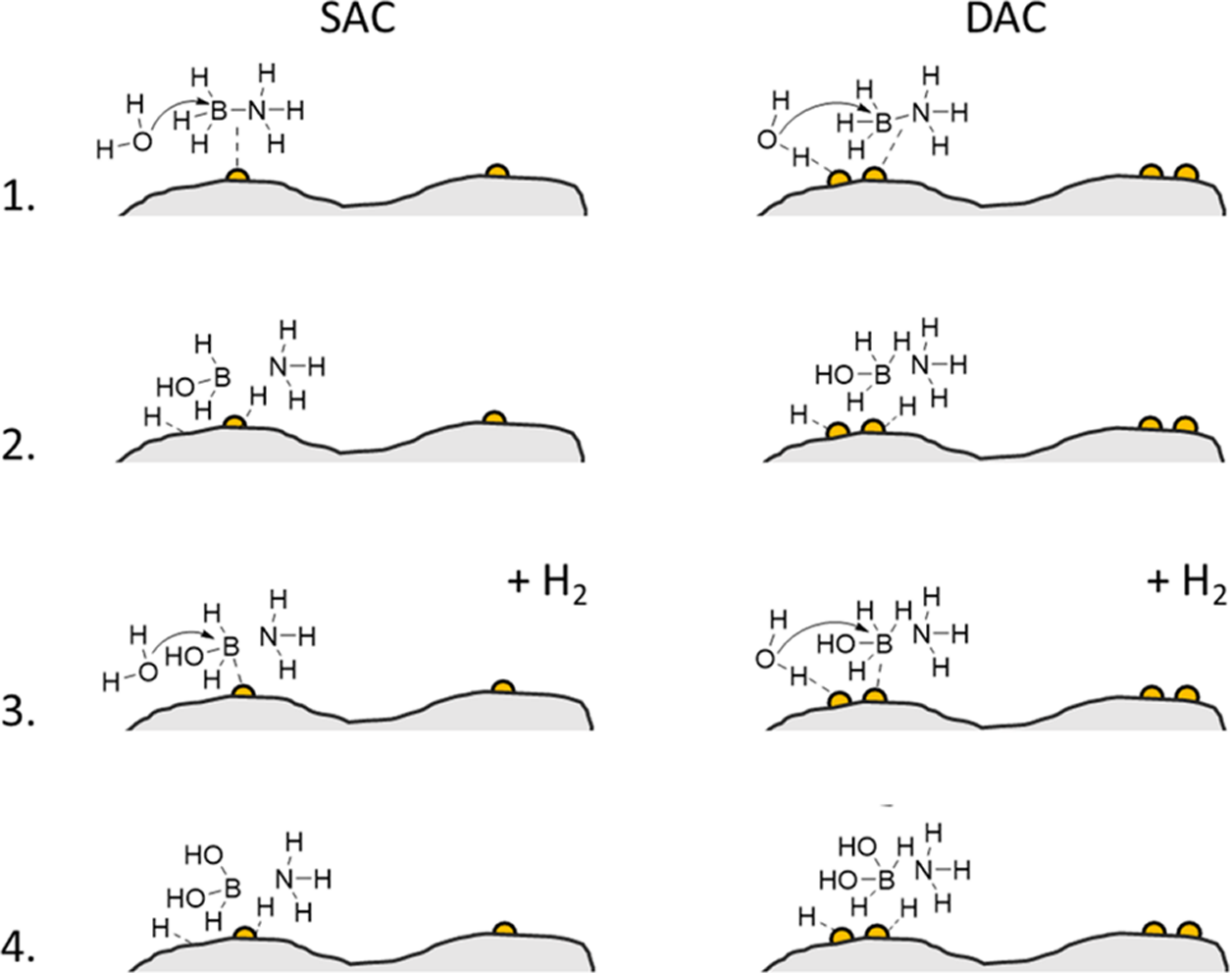
Ammonia borane hydrolysis and activation scheme for SACs (left) and DACs (right). (1) Adsorption of water and ammonia borane induces an S_N_2 reaction. (2) The B–N bond breaks at the transition state with the nucleophilic attack of oxygen. The adsorbed hydrogen atoms form a hydrogen molecule, which then leaves. (3) Adsorption of another water molecule in proximity to that of the intermediate. (4) Repeat of the process, forming another H_2_ molecule.

To understand the difference in catalytic activity, we first need to understand the meaning of the pre-exponential factors in both cases. The classic gas-phase explanation that assigns the pre-exponential factor as a “frequency factor” based on collisions and molecular cross sections does not apply here. Rather, the pre-exponential factor in such liquid–solid systems reflects the surface travel of species to/from the active site and their subsequent organization around the active site.^53^ Once an NH_3_BH_3_ molecule reaches the active site, it needs to react with a molecule of water. We know that each Pt site is most likely occupied by water, given the large difference in concentration ([H_2_O] = 55.5 M and [NH_3_BH_3_] = 0.1 M) and that both substrates are required at the active site for a successful reaction.^14^ Thus, in both cases, the water surrounds the Pt active site. However, in the case of the SAC, there is no additional adjacent site for activating this water, and therefore, practically no reaction is observed at lower temperatures because the surrounding water does not have enough energy to react. As the reaction vessel is heated, a larger fraction of the water molecules have the energy needed for crossing the barrier for ammonia borane hydrolysis. Once the temperature reaches 43 °C, the reaction proceeds readily via the S_N_2 pathway (see Figure 4).

For the DAC, things are different. When the NH_3_BH_3_ molecules adsorb at one Pt site, there is already an activated water molecule adsorbed at the adjacent Pt site (the chance that both sites are occupied simultaneously by ammonia borane is <1:250,000, based on their respective concentrations). This water molecule already has the energy needed for the reaction, and therefore, the reaction proceeds via the same S_N_2 pathway, already at 20 °C.

We hypothesize that the Pt atoms of the DAC work cooperatively: one Pt atom of the DAC activates H_2_O while the other one bounds to NH_3_BH_3_ (Figure 4). The SAC, however, cannot benefit from this because its Pt atoms are too far away from each other (a simple back-of-the-envelope calculation shows that to stabilize the O–B–N transition structure that corresponds to the S_N_2 mechanism, the Pt atoms must be <6 Å apart). Thus, the proximity of two active sites in the DAC causes the difference in activity. This is in line with the conclusion of Jin et al. that 1.2 nm was the upper limit for such proximity effects.^37^ The additional platinum atom can also be seen as a promoter. Such promoters can increase the activity but also decrease the selectivity through poisoning of the intermediates.^13,54^ We can compare this situation to that of the ORR, where substrate activation is considered difficult on SACs, usually leading to H_2_O_2_ formation instead of water. Zhang et al. showed that using DACs lowered the ORR activation barrier for Fe–Co DACs compared to either Fe or Co SACs.^55^

**Figure 5:**
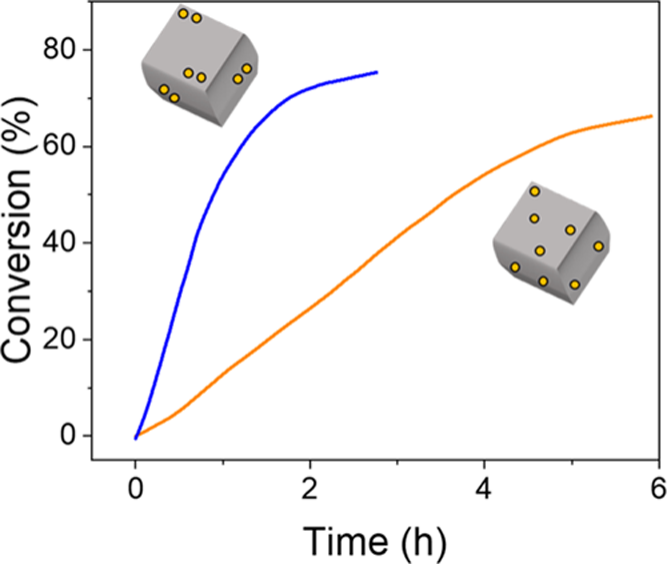
Isothermal hydrolysis studies of ammonia borane for the SAC and the DAC at 40 °C. Note the faster NH_3_BH_3_ conversion for the DAC compared with the SAC. Also, note the incomplete conversion for both runs.

To gain insight into the rate-determining step, we ran isothermal experiments at 40 °C (Figure 5). In both cases, we see that the reaction rate is constant and then reduces gradually. This holds for both the SAC and the DAC, although the DAC is 3 times as active. The linear behavior at the early stages of the reaction indicates a zero-order in ammonia borane, in agreement with published results that show that the water O–H bond scission is rate-determining.^48^

Interestingly, neither catalyst reached full conversion. This can be caused by poisoning or Pt leaching. We show that the former is most likely the dominant factor, by buildup of ammonium metaborate salts on the platinum active sites (see FTIR data in Figure S15a, where the spent catalyst shows a similar spectrum to that of NH_4_BO_2_). Such coating is common in borohydride hydrolysis systems.^56^ This also explains the differences between the CO-DRIFTS of the pristine and spent catalysts (Figures 1b and S15b). Nevertheless, the support retains its structure (see SEM in Figure S15c,d), so the metal–support interaction should not change during the reaction.

## Conclusions

Platinum dual-atom catalysts (DACs) outperform their single-atom analogues (SACs) in ammonia borane hydrolysis. Kinetic studies of the SACs show two temperature-dependent regions, with a transition point at 43 °C, while the DAC shows only one regime. The rate of the SAC is dependent on the surface travel of NH_3_BH_3_ to the active site—a thermal barrier that is overcome above 43 °C. This is not the case for the DAC, which shows cooperativity at its dual-atom site: one Pt atom activates H_2_O while the other binds NH_3_BH_3_. Importantly, DACs enhance the reaction by forming the protic and hydridic hydrogen in proximity (reflected in a higher pre-exponential factor) and enhanced water activation (reflected in the reaction starting at a lower temperature). We find that the rate-determining step is the water O–H bond scission, in agreement with previous work. This is further affirmed by high-precision kinetic studies that allow us to separate the water activation and hydrogen spillover effects. Using ammonia borane hydrolysis as a benchmark reaction gives additional insight into the workings of platinum SACs and DACs, above that which is obtained from traditional CO oxidation studies. Namely, we can quantify better the proximity effects of the platinum atoms in DACs on cerium oxide due to the low background activity of CeO_2_ in ammonia borane hydrolysis. We therefore suggest to other researchers in this field to study such proximity effects with other reactions in the future.

## Experimental Section

### Materials

All chemicals were purchased from commercial sources and used as received. Specifically, ammonia borane (technical grade, 90%) was obtained from Sigma-Aldrich, Ce(NO_3_)_3_·6H_2_O (grade, 99%) was obtained from Sigma-Aldrich, NaOH (grade, 99.99%) and KBr (grade, 99%) were obtained from VWR International. H_2_PtCl_6_·6H_2_O was obtained from Sigma-Aldrich (grade, 98.7%). [Pt_2_I_2_(H_2_NCH_2_CH_2_NH_2_)_2_](NO_3_)_2_ was obtained from Strem Chemicals (grade, 99%). All water used was demineralized water, which was deionized by the Milli-Q technique. It had a resistivity of 18.2 Ω cm.

### Instrumentation

Powder X-ray diffraction (pXRD) patterns were obtained with a MiniFlex II diffractometer using Ni-filtered Cu Kα radiation (1.541874 Å), ranging from 20 to 90°. The X-ray tube was operated at 30 kV and 15 mA, with a 2.5° step and 1 s dwell time. Scanning electron microscopy (SEM) was performed on a FEI Verios 460, which was equipped with an Oxford Xmax 80 mm^2^ silicon drift detector. A 5 kV accelerating voltage was used. The sample was ground with a pestle and mortar and loaded on a monocrystalline silicon holder. CO-DRIFTS measurements were performed on a Thermo Fischer Nicolet iS-50 Fourier transform infrared spectrometer equipped with a mercury–cadmium–telluride (MCT) detector and a KBr beamsplitter. A 4 cm^–1^ resolution was used in the 800–4000 cm^–1^ regime. The sample cup was loaded with the catalyst in a high vacuum chamber (HVC-DRM-5). This was placed in a Harrick praying mantis. The temperature in the high vacuum chamber was controlled with a Harrick temperature control unit (ATK-023-4). Samples were pretreated with a high-temperature vacuum (*T* = 200 °C, ρ = 10^–9^ bar) to ensure desorption of O_2_ and H_2_O, cooled down to room temperature, and subsequently treated with CO until the chamber was saturated. The chamber was then depressurized to 10^–9^ bar, ensuring no residual CO, and then CO-adsorption spectra were collected. HAADF-STEM images were obtained with an aberration-corrected transmission electron microscope JEOL JEM-ARM300F2 GRAND ARM 2 instrument, which was equipped with a high-angle annular dark field (HAADF) detector and an energy-dispersive X-ray (EDX) spectroscopy detector. The setup was operated at 300 kV and offers a spatial resolution of ≤60 pm in both TEM and STEM, resulting in atomic-resolution imaging of the samples.

XPS measurements were performed on a Scienta Omicron HiPP-3 analyzer operated in Swift acceleration mode and a monochromatic Al Kα source. The base pressure was about 2 × 10^–9^ mbar, and the operating pressure was about 5 × 10^–9^ mbar. Survey and high-resolution spectra were acquired at pass energies of 500 and 100 eV, respectively. Prior to data processing, the binding energies were calibrated with adventitious C 1s (284.8 eV) as the reference. A low binding-energy shoulder was detected for the Ce 3d, O 1s, C 1s, and Pt 4f peaks. This component is attributed to peak distortion caused by strong charge accumulation at the surface. XPS peak fitting was performed using KolXPD software from Kolibrik, employing Shirley background and Voigt functions for the individual components. Ce M_5,4_-edge and O K-edge X-ray absorption near edge structure (XANES) spectra were obtained at the X-ray magnetic circular dichroism (XMCD) beamline of Hefei Light Source (HLS). The Ce M_5,4_-edge data was fitted by standard Gaussian curves using multipeak fitting in Origin 2018.

CW X-band electron paramagnetic resonance (EPR) spectra were recorded in quartz tubes with a Bruker EMX-plus CW X-band spectrometer at room temperature. The effective *g* values were defined as the magnetic field strength at the maximum microwave absorption according to eq 2, where *g*_eff_ is the effective *g*-value, *h* is Planck’s constant (4.135 × 10^–15^ eV s), *v* is the microwave frequency of the spectrometer (9.643 GHz), μ_B_ is the Bohr magneton (5.788 × 10^–5^ eV T^–1^), and *B* is the applied magnetic field at the microwave absorption maximum in Tesla.
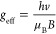
2

Metal loadings were determined by inductively coupled plasma optical emission spectrometry (ICP-OES). Analysis was done by Mikroanalytisches Laboratorium Kolbe, Oberhausen, Germany. The catalyst matrix was destroyed by microwave digestion and subsequently analyzed with a Spectro Arcos analyzer of Spectro, which maintains a standard error of ±1.5 ppm.

Thermogravimetric analysis and differential scanning calorimetry (TGA-DSC) were carried out using a NETZSCH Jupiter STA 449F3 instrument. The measurements were performed under a flow of air (20 mL min^–1^) in the temperature range 30–800 °C, using a scan rate of 5 °C min^–1^. Approximately 10 mg of the sample was analyzed.

### Procedure for Catalyst Preparation

CeO_2_ nanocubes were synthesized according to a hydrothermal treatment by Over and co-workers.^44^ The total reaction volume (and that of other precursors) was scaled to 90 mL and put in a 100 mL stainless steel autoclave with a Teflon insert. Then, 200 mg CeO_2_ nanocubes were transferred to a 10 mL RBF. This was suspended in 10 mL H_2_O by ultrasonication for 30 min. An appropriate amount of H_2_PtCl_6_·6H_2_O or Di-μ-iodobis(ethylenediamine)diplatinum(II) nitrate was dissolved in H_2_O and then added dropwise to the suspension, and this was stirred at room temperature for 1h. The product was stepwise dried using a rotary evaporator (i) 150 rpm, 55 mbar, 40 °C, 30 min, (ii) 150 rpm, 50 mbar, 40 °C, 30 min, 150 rpm, and (iii) 150 rpm, 45 mbar, 40 °C, 30 min. It was then further dried at 10 mbar, 40 °C. The product was then calcined at 500 °C for 2h with a ramp rate of 5 °C/min under static air.

### Procedure for Catalyst Testing

Approximately 10 mg of the catalyst was suspended in 8 mL of ultrapure H_2_O by ultrasonication in a 10 mL vial. Then, 0.4 mL of 2 M NH_3_BH_3_ solution in H_2_O (for nonisothermal experiments) and 0.4 mL of 0.2 M NH_3_BH_3_ solution in H_2_O (for isothermal experiments) were added with a syringe (1.0 mL), which was equipped with a glass capillary (Ø = 0.32 mm, *l* = 15 cm). Nonisothermal salt experiments were done in a 1:2 Pt:X (X = Cl^–^ or I^–^) molar ratio pre-prepared solution in 8 mL H_2_O. The reaction kinetics were monitored with a custom-built bubble detector.^57^ The reactor was continuously stirred with a stirring bar (8.0 × 3.0 mm^2^), and it operated at 1 atm. Nitrogen was purged through the device before the addition of the ammonia borane solution. Data processing of the results was done as described elsewhere.^53^ The size and amount of the H_2_ bubbles were recorded during the reaction by the time-dependent interruption of the laser beam by a bubble. The detection cell was filled with hexadecane. The temperature inside the reactor was recorded as well and heated with 2 °C/min for nonisothermal kinetic studies (starting from 5 °C, cooled with an external icebath, until 80 °C) after addition of the ammonia borane solution. Gas expansion corrections of the headspace of the reactor and an increased vapor pressure of the used solvent at elevated temperatures were corrected for in all experiments. All experiments were performed in duplicate.

### Computational Methods

Periodic spin-polarized density functional theory (DFT) calculations were performed using the Vienna Ab initio Simulation Package (VASP).^58,59^ The exchange–correlation energy was calculated with the PBE functional.^60^ For an accurate description of the highly localized Ce 4f-orbitals, DFT + *U* calculations^61^ were performed with a *U* parameter of 4.5 eV applied to the Ce 4f states, in line with the previous studies.^40,62,63^ The surfaces were cut from a bulk cubic (Fm3m) CeO_2_ structure with a lattice parameter of 5.49 Å, optimized with the PBE + *U* formalism. The calculated lattice parameter is in line with previously determined experimental and DFT calculated values.^40,62,63^ The electron–ion interactions were modeled using the projector-augmented wave (PAW)^64^ method, with a plane wave cutoff energy of 400 eV. A 15 Å vacuum region was placed above the slabs to avoid electronic interactions in the *z*-direction. For the Brillouin-zone integration, a γ point was used due to the large size of the unit cell. CeO_2_ surface was modeled with a nondefective, pristine, CeO_2_(111) slab with 9 atomic layers, consisting of 48 Ce and 96 O atoms. The (111) facet was used as it is thermodynamically the most stable facet of CeO_2_ and was also observed in experimental studies.^65^ During the optimizations, the atomic positions of the lowermost 3 layers were kept fixed to represent the bulk structure. Increasing the slab thickness above 9 layers resulted in less than a 0.1% change in the CO stretching frequency. The optimizations regarding Pt-doped CeO_2_(111) surfaces and CO adsorbed on Pt*_X_*–CeO_2_ were performed on unit cells of the slabs with p(4 × 4) periodicity in the *x* and *y* directions. The size of the unit cell was chosen to eliminate the coverage effects that could influence the CO vibrational frequency due to resonance between adsorbed CO on neighboring unit cells. Single (Pt_1_–CeO_2_) and double (Pt_2_–CeO_2_) Pt atom catalysts on CeO_2_(111) were modeled by substituting Ce atoms at the topmost layer by Pt, as it was mentioned that substitution of Pt by Ce is more energetically favorable than the adsorption of Pt in a recent theoretical investigation.^63^ CO-adsorption structures were calculated on the optimized Pt*_X_*–CeO_2_ surfaces. CO-adsorption structures were confirmed to be energy minima based on having no imaginary frequencies indicated by vibrational analysis. During vibrational frequency analysis, the atoms were displaced from their equilibrium positions by 0.015 Å. C–O stretching frequencies were reported based on the vibrational analysis of adsorbed CO on Pt*_X_*–CeO_2_(111) surfaces.

## Supporting Information Available

Additional characterization of the synthesized and spent catalysts and the DFT calculations with the coordinates of the atoms of the different models. (PDF)
