# Supplementary material for: Bottom-Up Synthesis of Platinum Dual-Atom Catalysts on Cerium Oxide
Source: ACS Catal. 2024 Jun 17;14(13):9850–9. doi: 10.1021/acscatal.4c01840 (PMC11232020; doi:10.1021/acscatal.4c01840)
Supplement: Supplementary file 1 — cs4c01840_si_002.pdf [file cs4c01840_si_002.pdf]

Supporting information for:

Bottom-up synthesis of platinum dual-atom catalysts on cerium oxide

Martijn J. Mekkering[a], Petrus C. M. Laan[a], Alessandro Troglia[b], Roland Bliem[b], Ali C. Kizilkaya[a,c], Gadi Rothenberg[a*], and Ning Yan [a,d*]

[a] Van 't Hoff Institute for Molecular Sciences, University of Amsterdam, Science Park 904, 1098 XH Amsterdam, The Netherlands.

[b] Advanced Research Center for Nanolithography (ARCNL), Science Park 106, 1098XG Amsterdam, The Netherlands.

[c] Department of Chemical Engineering, Izmir Institute of Technology, 35430, Urla, Izmir, Türkiye.

[d] Key Laboratory of Artificial Micro- and Nano-Structures of Ministry of Education, School of Physics and Technology, Wuhan University, Wuhan, 430072, China.

E-mail: g.rothenberg@uva.nl ; ning.yan@whu.edu.cn

Table of Contents

Cerium oxide support characterization S2

Decomposition of the DAC precursor S3

Physical characterization of CeO_2, Pt_1/CeO_2 (SAC) and Pt_2 /CeO_2 (DAC) S3

Analysis of spent catalyst S12

Ammonia borane hydrolysis experiments S12

Calculation of occupation of SAC and DAC sites S13

DFT simulations S13

Coordinates of the different model systems S14

References S35

1. characterization

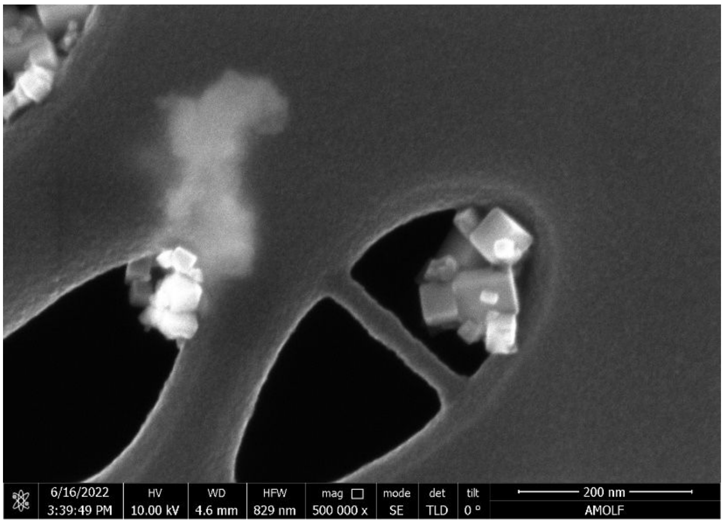

Figure S1 – SEM image of the CeO_2 cubes.

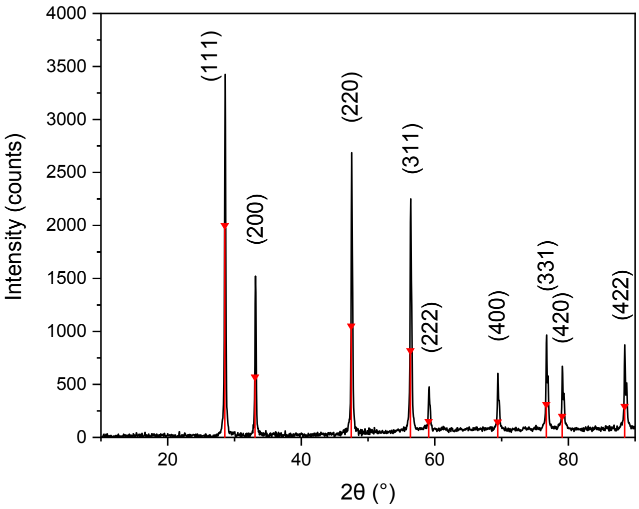

Figure S2 – pXRD pattern of the CeO2 cubes. The diffraction lines match literature (red). [1]

2. Decomposition of the DAC precursor

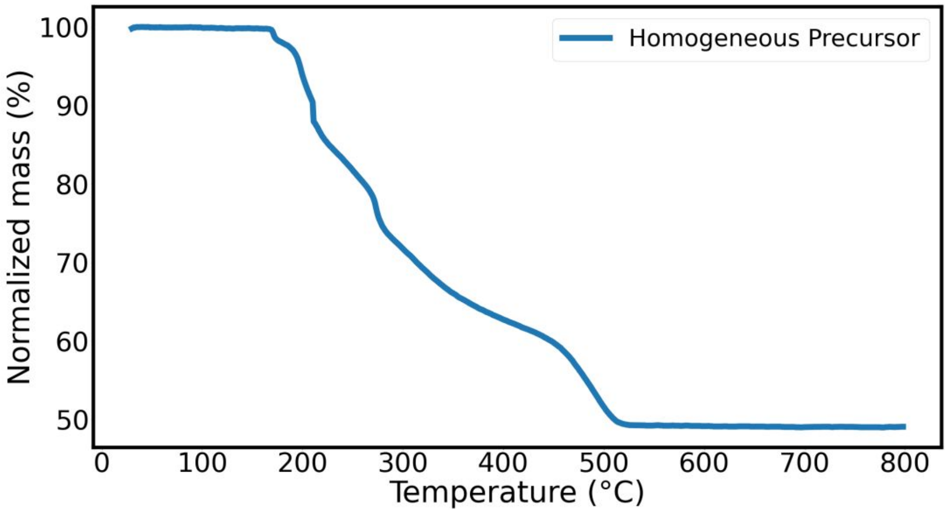

Figure S3 – TGA trace of the double atomic precursor $[Pt_2I_2(H_2NCH_2CH_2NH_2)_2](NO_3)_2$.

3. Physical characterisation of CeO_2, Pt_1/CeO_2 (SAC) and Pt_2/CeO_2 (DAC)

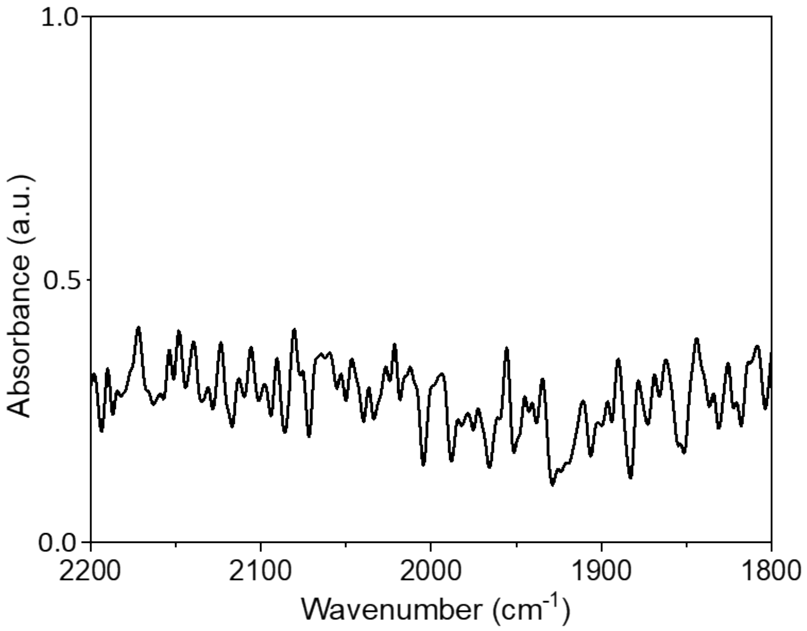

Figure S4 – CO-DRIFTS of the pristine CeO_2 support, showing no peak at around 2100 cm1^{-1}.

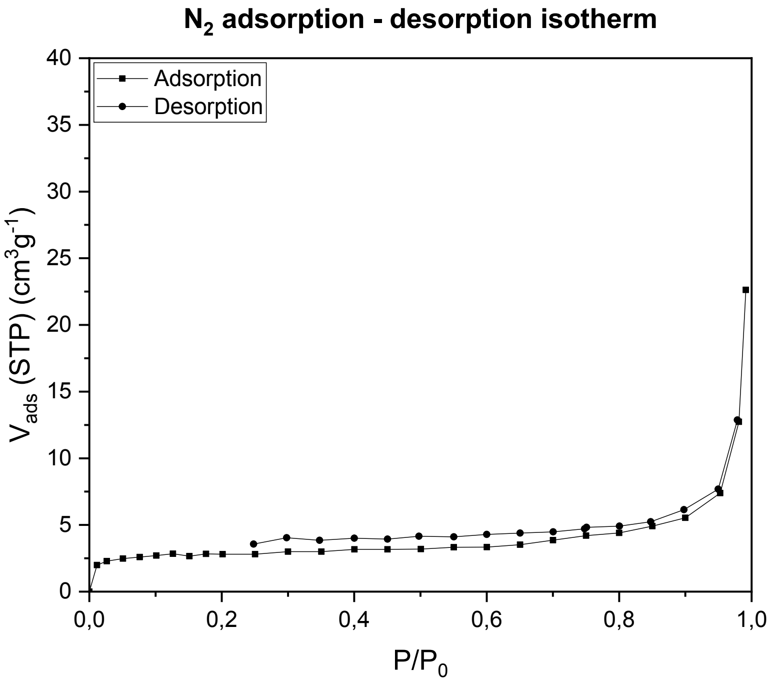

Figure S5 – N_2 adsorption plot of the CeO_2 cube. The BET surface area was 8 m^2/g.

The calculation of the interatomic distance is done according to the following formula:

$$D_{M-M}(nm) = sqrt\left(\frac{S_{BET}(nm^2/g)}{\frac{weight\ loading\ (\%)}{100\ (\%)} * \frac{1}{MW_{Pt}(g/mol)} * N_{AV}}\right)$$

Where $D_{M\text{-}M}$ is the distance of two platinum metals in the SAC, S_{BET} is the BET surface area, MW_{Pt} is the weight of one mol of platinum (197 g/mol) and N_{AV} is the Avogadro constant.

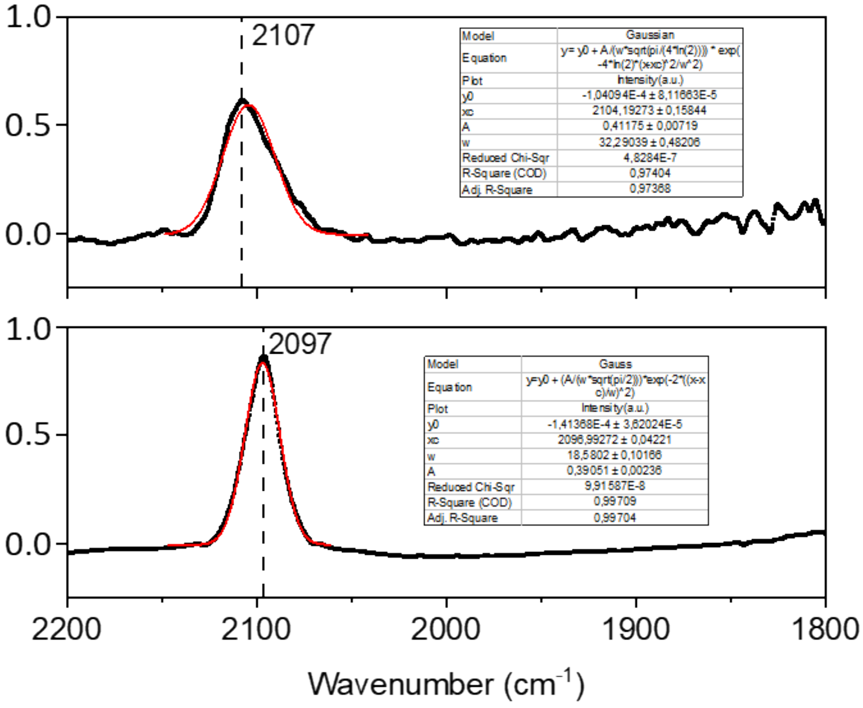

Figure S6 – Fitted CO-DRIFTS spectra of the Pt SAC (above) and DAC (below). Both are fitted with a single component.

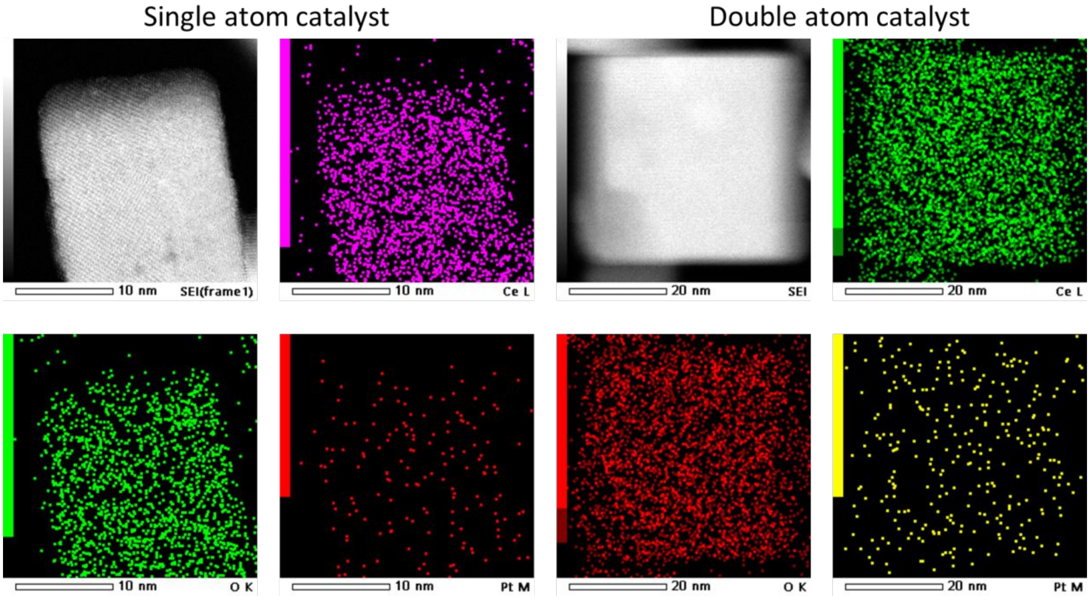

Figure S7 – HAADF-STEM images of the as-synthesized Pt_1 and Pt_2 CeO_2 cubes. No clustering or isolated atoms were observed.

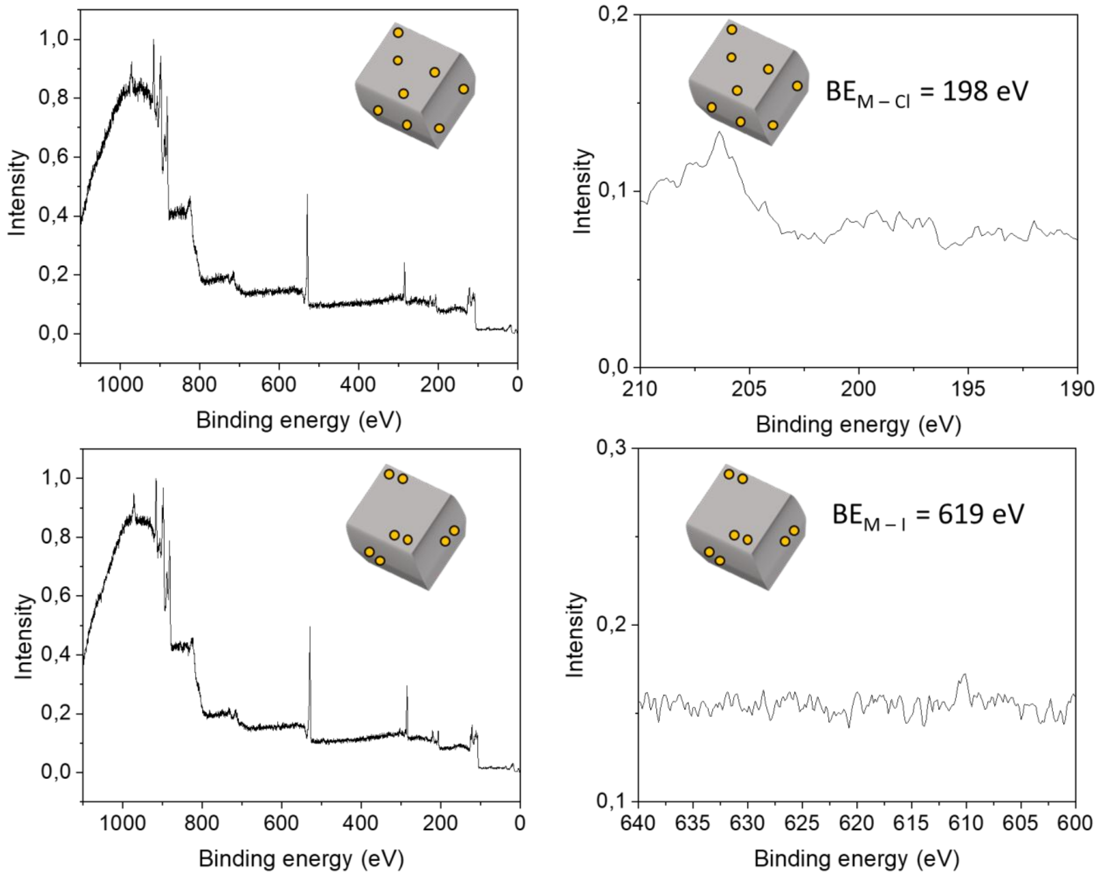

Figure S8 – XP survey spectrum of the SAC (above) and DAC (below) and zoom in at the M-Cl and M-I peaks at the corresponding binding energies. No deposition of both Cl^- and I^- was observed in the SAC and DAC, respectively, by comparing the reference binding energies of metal iodides[2] and metal chlorides[3]. The peak at 207 eV is the electron in the Ce $4p_{3/2}$ orbital.

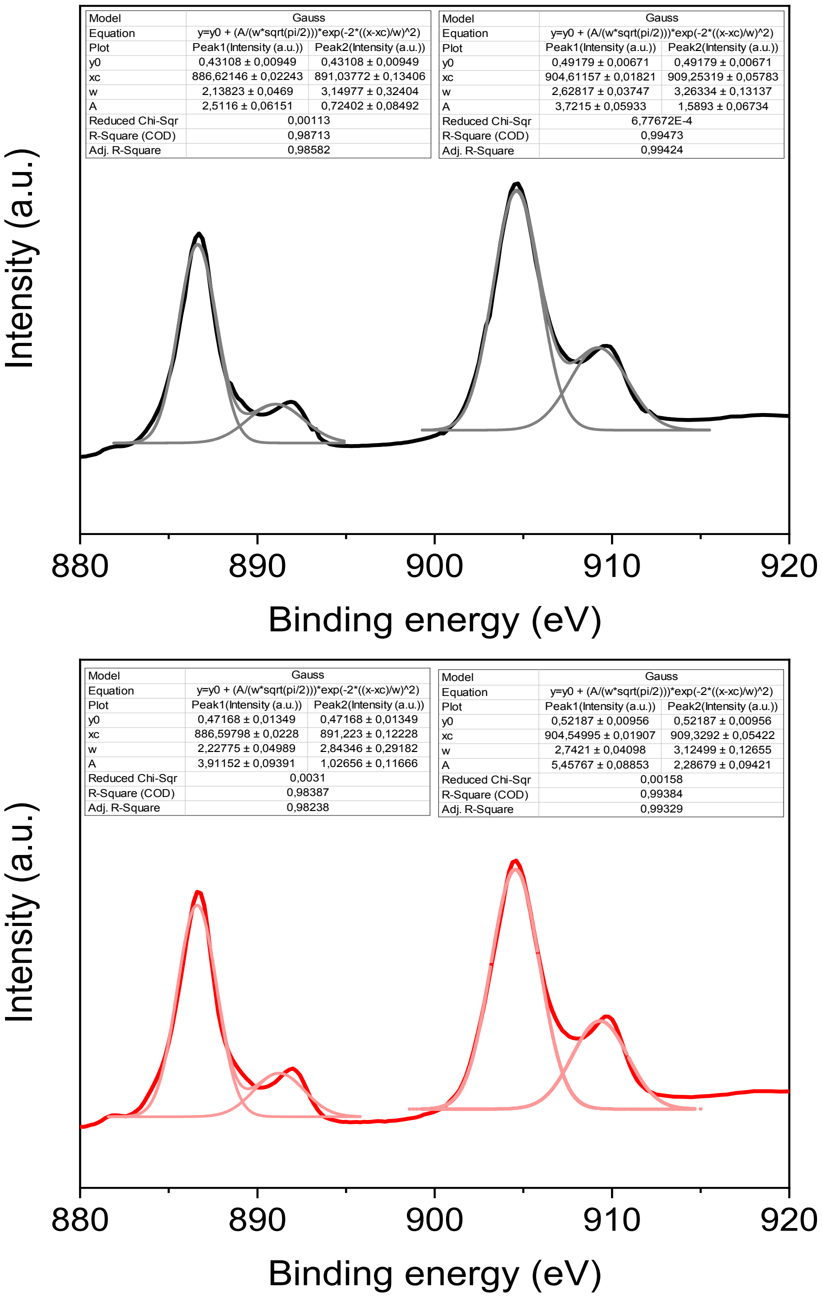

Figure S9 – Fitted XAS spectra of polycrystalline CeO_2 (black) and cubic CeO_2 (red). The inserts are the fits of the two components in each peak of the respective peak.

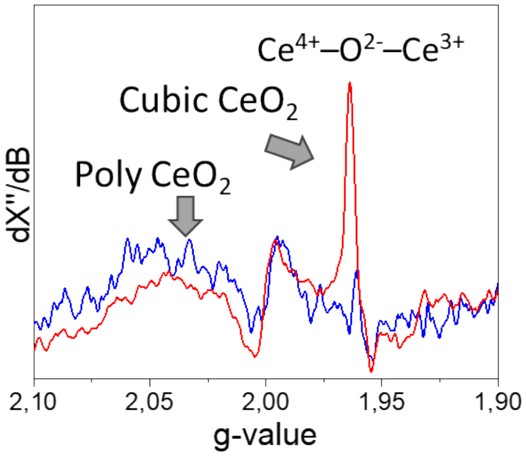

Figure S10 – EPR measurements on the cerium oxide support.

In Figure S10 we show the presence of the Ce^{4+}–O^{2-}–Ce^{3+} site at a g-value of 1.96.[4]

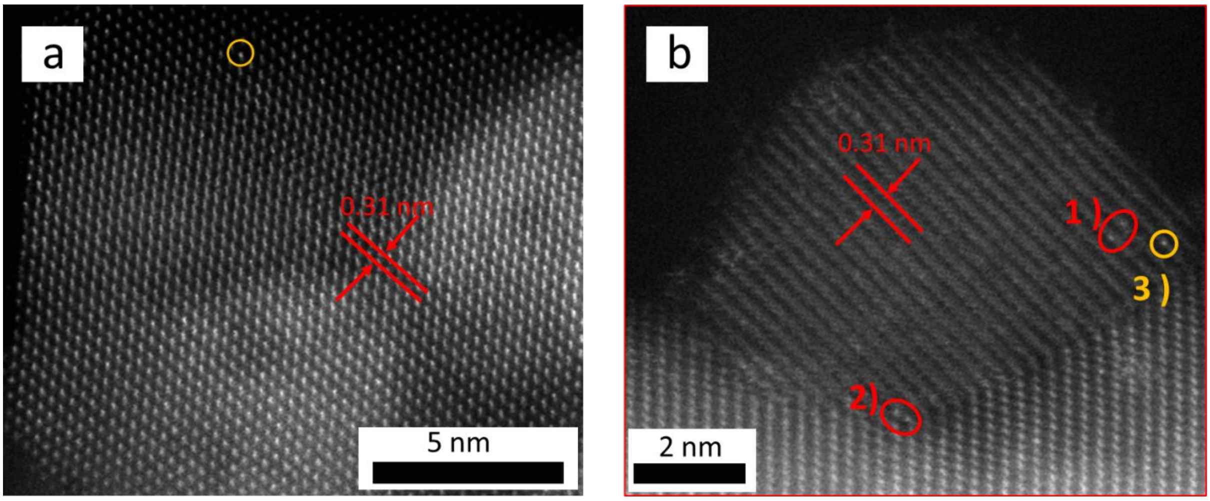

Figure S11 – AC-HR-TEM images of the SAC (a) and DAC (b) without the rectangles as presented in the main text.

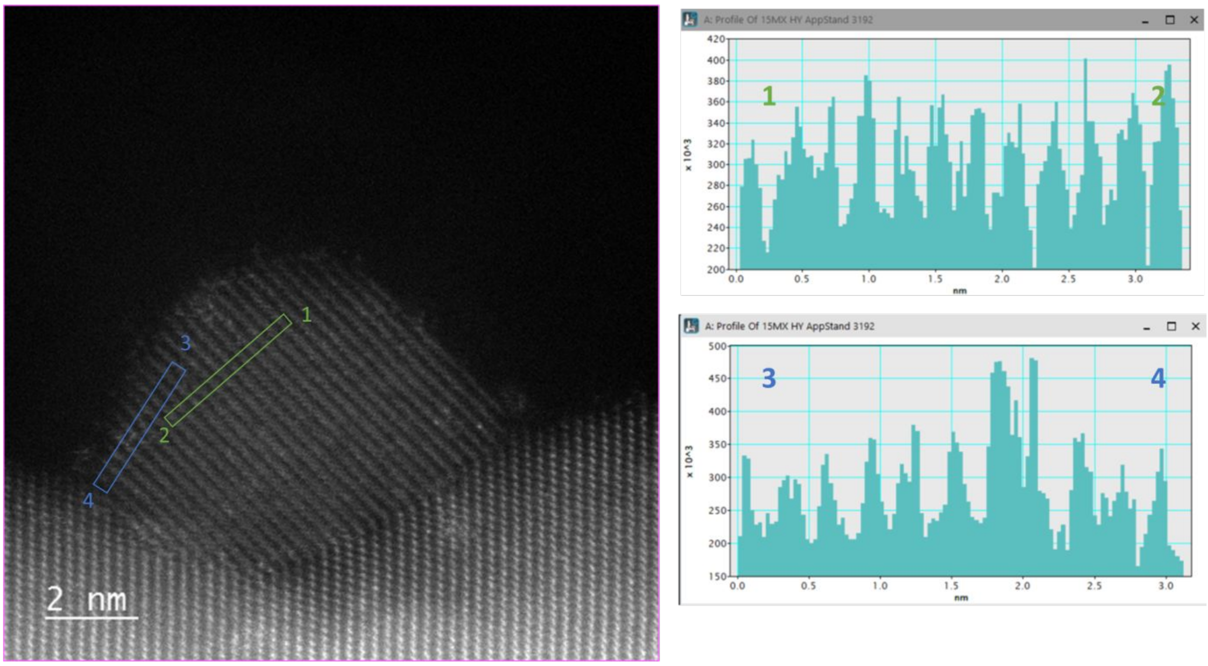

Figure S12 Additional line scans on the DAC as presented in the main text, Figure 2.

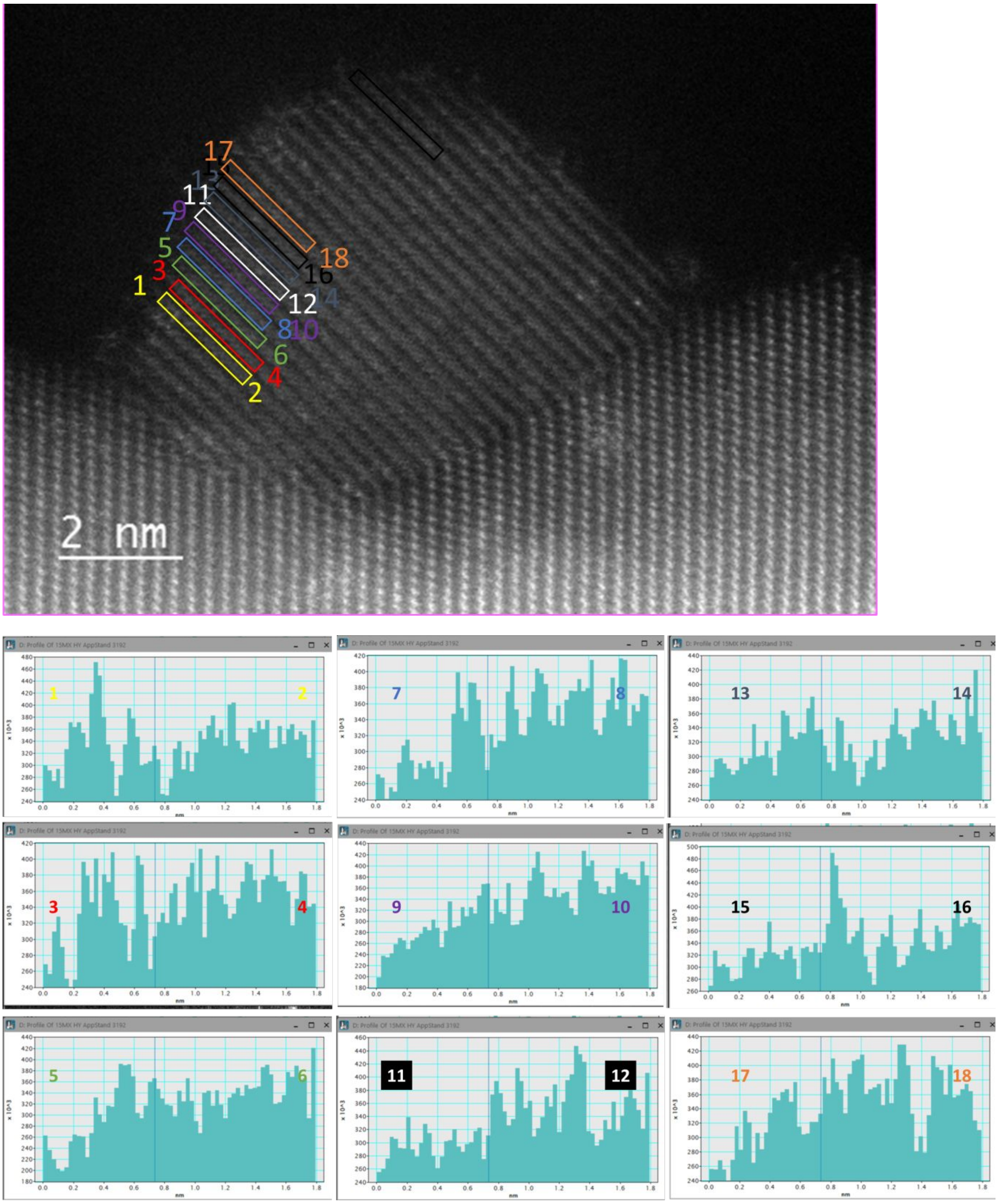

Figure S13 – Additional line scans on the DAC as presented in the main text, Figure 2.

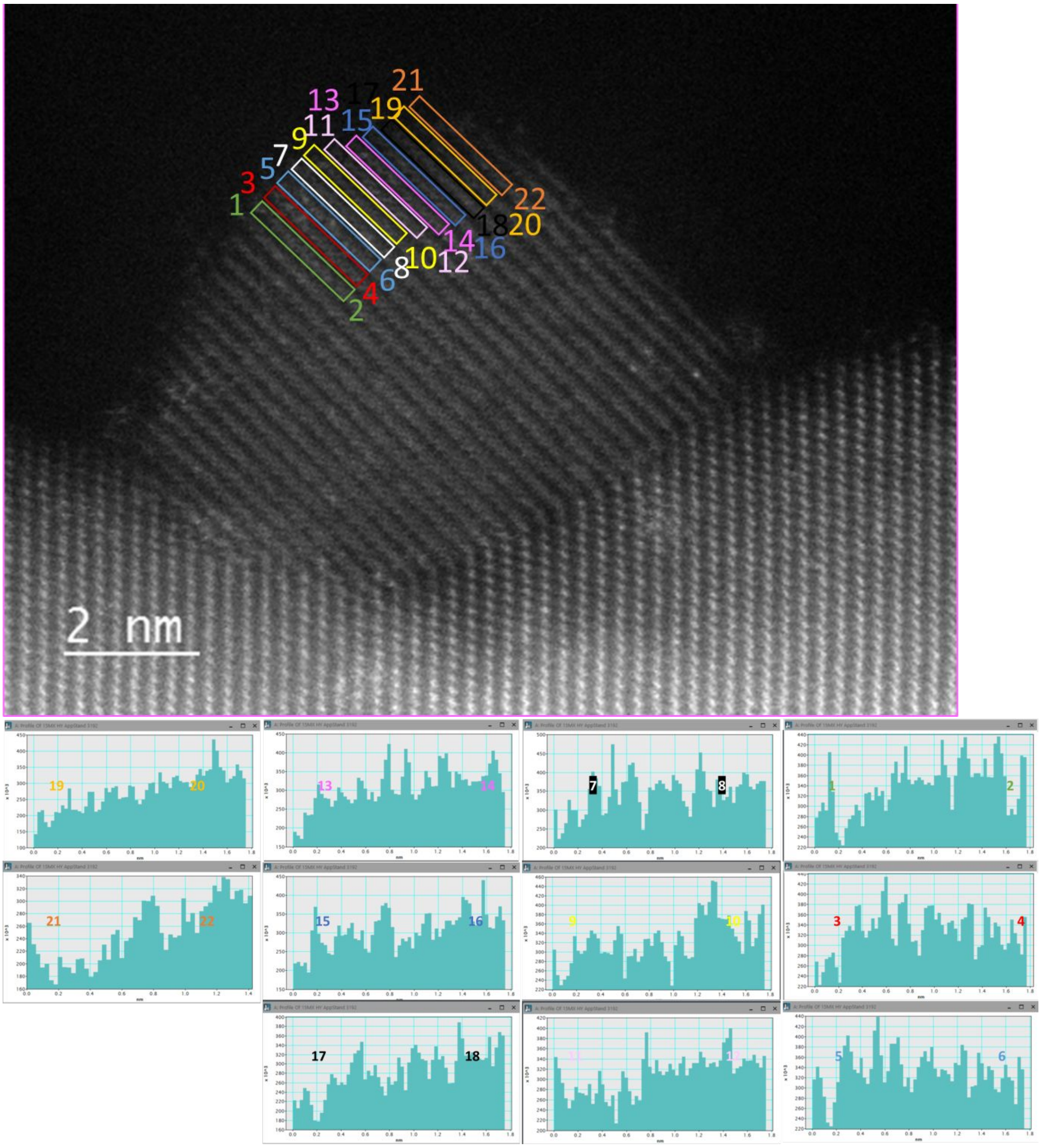

Figure S14 – Additional line scans on the DAC as presented in the main text, Figure 2.

4. Analysis of spent catalyst

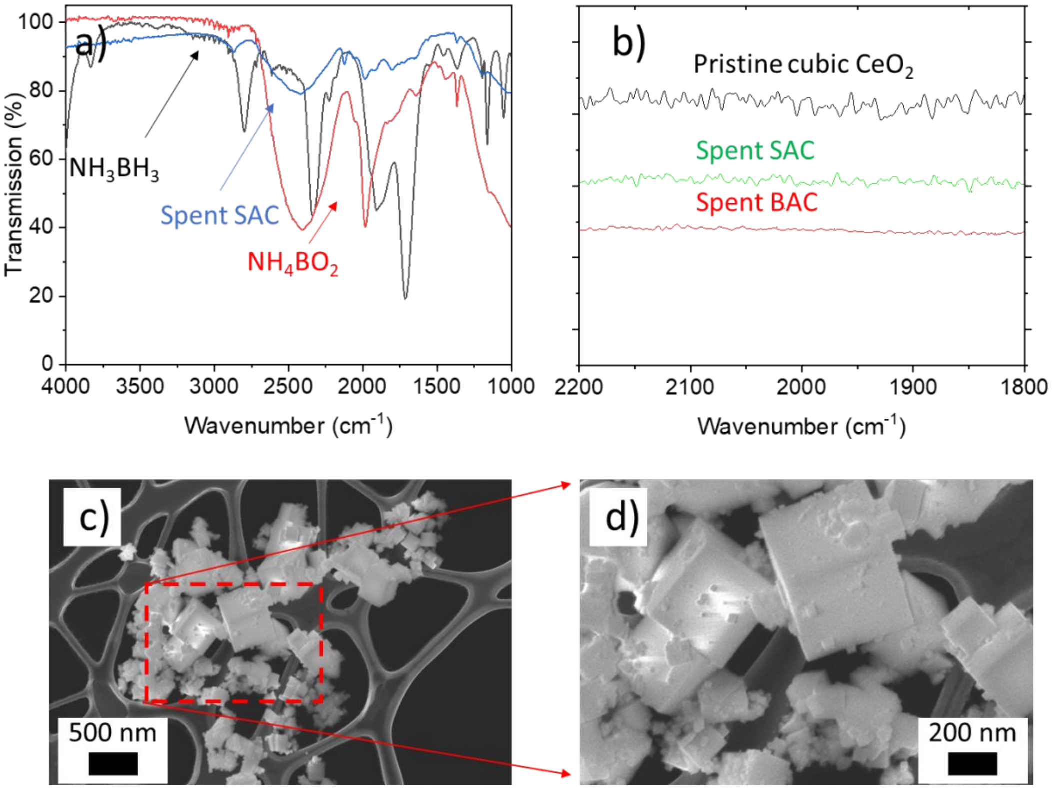

Figure S15 – (a) IR-spectra of NH_3BH_3, the spent catalyst powder and NH_4BO_2 showing deposition of NH_4BO_2 on the catalysts. (b) CO-DRIFTS spectra after the reaction showing no detectable Pt-species. (c) SEM-image of the spent catalyst showing that the CeO_2 cube retained its structure during catalysis. Notice the surface deposition around the particle (see zoom-in in (d)), which is not present on the CeO_2 particles before the reaction (c.f. Figure S1).

5. Ammonia borane hydrolysis experiments

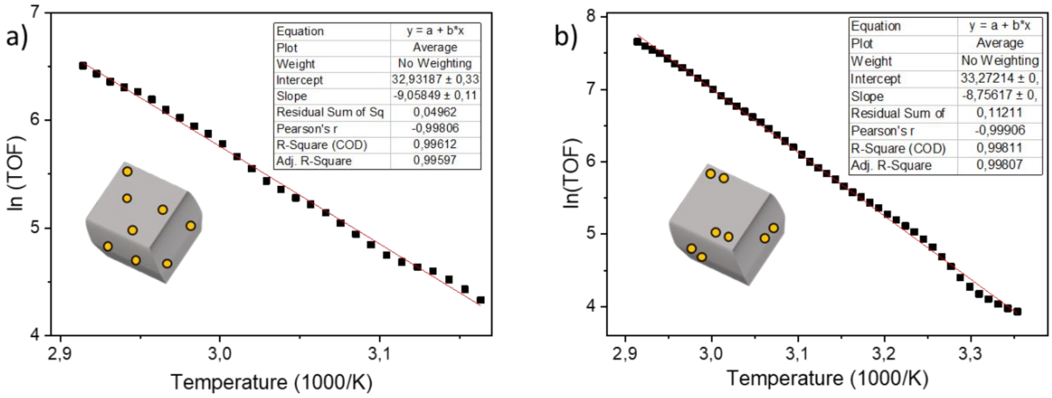

Figure S16 – Fittings of the ammonia borane hydrolysis experiments indicating the slope and intercept of the SAC (a) and the DAC (b). Note that the slope of the fit of the double-atom catalysed reaction is slightly lower and the pre-exponential factor is 4 times higher (after taking the exponential as the intercept is the natural logarithm of the pre-exponential factor).

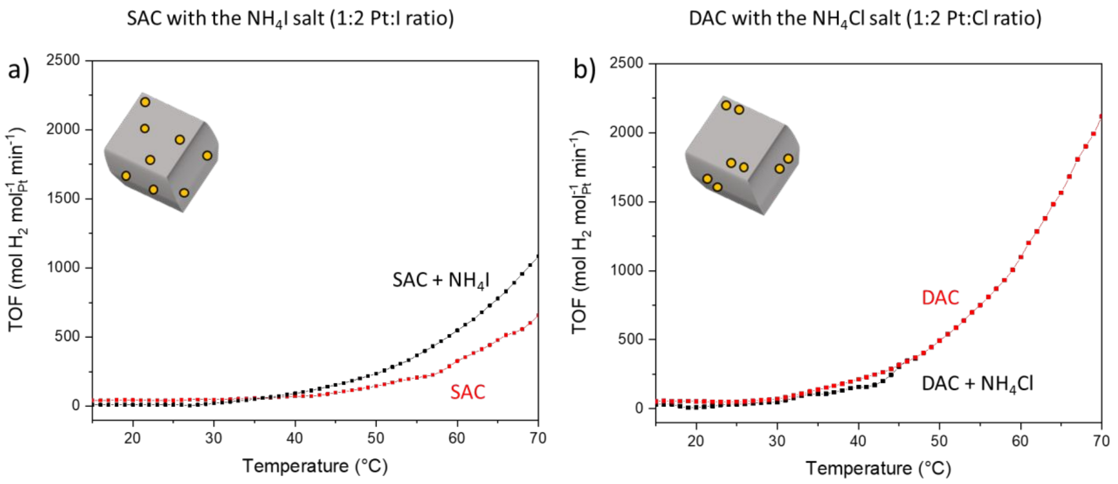

Figure S17 – Experiments of the added salt to either the SAC (a) or the DAC (b) in a 1:2 Pt:X ratio. Note the very similar performance for the SAC. For the DAC a water bubble from the solution entered the detection cell due to evaporation at T = 47 °C, which affected the bubble formation.

6. Calculation of occupation of SAC and DAC sites

Given the experimental conditions mentioned in the experimental details, 8.4 mL H_2O was available in the reaction mixture as well as 0.8 mmol NH_3BH_3. Thus (8.4 mL H_2O × 55.5 M H_2O in H_2O=) 466.6 mmol H_2O was available with each 0.8 mmol NH_3BH_3. 466.6 / 0.8 ≈ 580.

Then calculating the ammonia borane occupation was 1/580 * 100% = 0.17% and the adsorption of ammonia borane at DAC pair is 0.17%*2 = 0.34%.

The adsorption of two ammonia borane molecules was 0.17% × 0.17% = 0.0003%.

7. DFT Simulations.

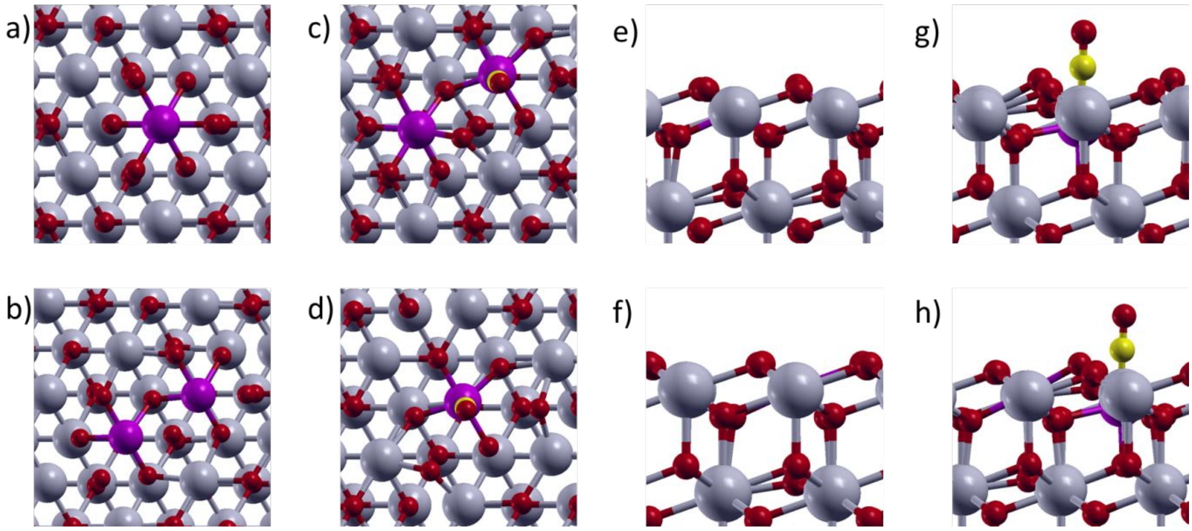

Figure S18 – Optimized structure of the Pt_x-CeO_2 models (a,b; top view – e,f; side view) and models where CO (yellow carbon atom and red oxygen atom) is adsorbed on the platinum (purple) site (c,d; top view – g,h; side view) of the SAC and DAC, respectively.

Tabel S1 – Bond lengths (Å) on pristine CeO_2, Pt_X–CeO_2(111) and COad–Pt_X–CeO_2

	CeO_2	Pt_1-CeO_2	COad–Pt_1–CeO_2	Pt_2–CeO_2	COad–Pt_2–CeO_2
Ce-Ce	3.88	3.81	3.72	3.81	3.72
Ce-O	2.37	2.41	2.51	2.49	2.51
Ce-Pt	NA	3.8	3.99	3.7	3.86
Pt-O	NA	2.18	2.08	2.13	2.07
Pt-Pt	NA	NA	NA	3.62	3.83
Pt-C	NA	NA	1.87	NA	1.87
C-O	NA	NA	1.15	NA	1.15

8. Coordinates of the different model systems

Pt_1@CeO_2

```
System
   1.00000000000000
    13.4388805250000001   -7.7589412890000000    0.0000000000000000
     0.0000000000000000   15.5178825770000000    0.0000000000000000
     0.0000000000000000    0.0000000000000000   19.0058000000000007
   Ce   O    Pt
    47   96    1
Selective dynamics
Direct
  0.1488931311114570  0.1389316520428210  0.0621532900483004   F   F
F
  0.1488931311114570  0.3889316105262068  0.0621532900483004   F   F
F
  0.3988931213450186  0.1389316679259665  0.0621532900483004   F   F
F
  0.3988931213450186  0.3889316264093523  0.0621532900483004   F   F
F
  0.1488931311114570  0.6389316334513708  0.0621532900483004   F   F
F
  0.1488931311114570  0.8889316563765348  0.0621532900483004   F   F
F
  0.3988931213450186  0.6389316493345163  0.0621532900483004   F   F
F
  0.3988931213450186  0.8889316078178950  0.0621532900483004   F   F
F
  0.6488931115785803  0.1389316838091119  0.0621532900483004   F   F
F
  0.6488931115785803  0.3889316422924978  0.0621532900483004   F   F
F
  0.8988931018121420  0.1389316996922574  0.0621532900483004   F   F
F
```

0.8988931018121420 0.3889316581756432 0.0621532900483004 F F
F
0.6488931115785803 0.6389316007758765 0.0621532900483004 F F
F
0.6488931115785803 0.8889316237010405 0.0621532900483004 F F
F
0.8988931018121420 0.6389316166590220 0.0621532900483004 F F
F
0.8988931018121420 0.8889316395841860 0.0621532900483004 F F
F
0.3161600464368413 0.2230417291564891 0.2284185482512550 T T
T
0.8158000963568653 0.9717373676717422 0.2284292095455352 T T
T
0.0650323192019697 0.9718232441731300 0.2282996928875640 T T
T
0.8141166455539922 0.4738336264622330 0.2264554132986864 T T
T
0.0655634108380287 0.2222688141140145 0.2272579413505521 T T
T
0.8141424347843067 0.7192813920296720 0.2264337062272684 T T
T
0.0651559814136953 0.7220779452199384 0.2279813181511725 T T
T
0.3156128701499649 0.9724998154603874 0.2285286988833590 T T
T
0.8157661618776723 0.2229930655900685 0.2284238252114458 T T
T
0.0648484368426925 0.4720785403350484 0.2284242970035390 T T
T
0.3154865830426391 0.7220239600905827 0.2286457695698127 T T
T
0.5659407778206568 0.2229194013895518 0.2281090279581574 T T
T
0.5658879621353861 0.9724141184106762 0.2286542569109579 T T
T
0.3161060712436148 0.4720935855123652 0.2284344645188066 T T
T
0.5686475324046493 0.4738294892252786 0.2264714520148604 T T
T
0.5658970174922477 0.7219919310627575 0.2281183717270459 T T
T
0.9779336865867897 0.8006973444781549 0.3940841561560639 T T
T
0.4873079718068132 0.3101657524526391 0.3941631987296353 T T
T
0.2324190028623494 0.0555428140799257 0.3940096268689504 T T
T
0.2318074138921256 0.3053981310443852 0.3929845679569053 T T
T
0.2316795614595618 0.8053121207945192 0.3951204537366043 T T
T
0.9824912492294917 0.0560141875370943 0.3929537489151703 T T
T
0.7318270108160674 0.8006231642077372 0.3941672003458424 T T
T
0.9778182577291917 0.5561476450871110 0.3941268073940332 T T
T

0.4826012441590344	0.0562583258624568	0.3951425179529932	T	T	T
0.7320607406038590	0.0555022562939698	0.3940542431348256	T	T	T
0.2323524932785223	0.5558067658861000	0.3940440505955195	T	T	T
0.9824346355375067	0.3053738905182598	0.3929950156085526	T	T	T
0.4826146720740419	0.8052998722027136	0.3951617387434297	T	T	T
0.7317907348483891	0.3101320597477714	0.3941678963721822	T	T	T
0.4872852416623651	0.5561518074237554	0.3941748086440616	T	T	T
0.2323574964485423	0.0556675870769677	0.0209352503760487	T	T	T
0.2322264115820047	0.3055982715224701	0.0205499900030546	F	F	F
0.4822264762265220	0.0555983661277182	0.0205499900030546	F	F	F
0.4822264762265220	0.3055983246110969	0.0205499900030546	F	F	F
0.2322264115820047	0.5555982944476341	0.0205499900030546	F	F	F
0.2322264115820047	0.8055982529310199	0.0205499900030546	F	F	F
0.4822264762265220	0.5555982830944828	0.0205499900030546	F	F	F
0.4822264762265220	0.8055983060196468	0.0205499900030546	F	F	F
0.7322263920491281	0.0555982803635970	0.0205499900030546	F	F	F
0.7322263920491281	0.3055983032887610	0.0205499900030546	F	F	F
0.9822264566936454	0.0555983334522239	0.0205499900030546	F	F	F
0.9822264566936454	0.3055983563773879	0.0205499900030546	F	F	F
0.7322263920491281	0.5555982617721469	0.0205499900030546	F	F	F
0.7322263920491281	0.8055982846973109	0.0205499900030546	F	F	F
0.9822264566936454	0.5555983148607737	0.0205499900030546	F	F	F
0.9822264566936454	0.8055983377859306	0.0205499900030546	F	F	F
0.0655597762299465	0.9722648937330050	0.1037486977659441	F	F	F
0.0655597762299465	0.2222649538410764	0.1037486977659441	F	F	F
0.3155597664635081	0.9722649740579286	0.1037486977659441	F	F	F
0.3155597664635081	0.2222649697242218	0.1037486977659441	F	F	F
0.0655597762299465	0.4722649767662404	0.1037486977659441	F	F	F
0.0655597762299465	0.7222648708078410	0.1037486977659441	F	F	F

0.3155597664635081	0.4722649926493858	0.1037486977659441	F	F	F
0.3155597664635081	0.7222649511327646	0.1037486977659441	F	F	F
0.5655597566970698	0.9722649254992959	0.1037486977659441	F	F	F
0.5655597566970698	0.2222649211655821	0.1037486977659441	F	F	F
0.8155597469306315	0.9722649413824414	0.1037486977659441	F	F	F
0.8155597469306315	0.2222650014905128	0.1037486977659441	F	F	F
0.5655597566970698	0.4722649440907460	0.1037486977659441	F	F	F
0.5655597566970698	0.7222649025741319	0.1037486977659441	F	F	F
0.8155597469306315	0.4722649599738915	0.1037486977659441	F	F	F
0.8155597469306315	0.7222649184572774	0.1037486977659441	F	F	F
0.1489850286469476	0.8889614302570976	0.1855009523408668	F	F	F
0.1489263533744761	0.1389755932800339	0.1858052988771719	T	T	T
0.8994747563180150	0.8899532469398066	0.1851676688034376	T	T	T
0.9057877189220794	0.6423824870055032	0.1840272746757984	T	T	T
0.8989391692427524	0.1389487641973355	0.1858215436095610	T	T	T
0.8994169442590679	0.3884410797113750	0.1851465152988682	T	T	T
0.6492551283328765	0.1397754612911455	0.1863720588490771	T	T	T
0.3978939483470784	0.3884289690502180	0.1851763649309659	T	T	T
0.1488753891079352	0.3888985939512755	0.1858226438459349	T	T	T
0.1481315142392577	0.6386473706952055	0.1863597712563499	T	T	T
0.3994982096355601	0.1398468266437776	0.1863960612553772	T	T	T
0.6492230333310084	0.8884291867527522	0.1863764634727172	T	T	T
0.3994136090554229	0.6386454769110359	0.1863799320004347	T	T	T
0.6455245922360323	0.3821501700253608	0.1840351945577344	T	T	T
0.3989681600737315	0.8889420780063974	0.1866169333467242	T	T	T
0.6455340390325111	0.6423675935596450	0.1840543233272502	T	T	T
0.2322203547255592	0.0555851699416017	0.2709076748504618	T	T	T
0.9823814499082419	0.0557428145613450	0.2707138765855122	T	T	T
0.2308080817194221	0.8049589226299214	0.2716281421798080	T	T	T

```
  0.7322974738988368  0.5556599955398135  0.2449074260457040   T   T
T
  0.2320684598105460  0.3055249373618025  0.2707607507153422   T   T
T
  0.9795893963752950  0.8052918928558100  0.2723704643098891   T   T
T
  0.7349439282269271  0.8051482049888546  0.2726121049466762   T   T
T
  0.4827085267703030  0.5529678754334262  0.2726252375333266   T   T
T
  0.7323063733618180  0.0556149031952803  0.2709306835111055   T   T
T
  0.9822982251881094  0.3055144154161451  0.2707600035255448   T   T
T
  0.2322242335123379  0.5555644219413680  0.2709235024499130   T   T
T
  0.7349796715631052  0.3088182788896537  0.2726131586478321   T   T
T
  0.9791622375167061  0.5529907498455851  0.2725620136828429   T   T
T
  0.4828189839419955  0.3088342229863935  0.2726272682325085   T   T
T
  0.4830705248507255  0.0570898747903579  0.2716717536769432   T   T
T
  0.4829782201719781  0.8048991278261567  0.2716938775132027   T   T
T
  0.3155814670892670  0.2229214645223463  0.3526746741142606   T   T
T
  0.8037935471935108  0.6983660146943824  0.3509881344051165   T   T
T
  0.3155191161905020  0.7221271629315926  0.3522150074033017   T   T
T
  0.0650255355365689  0.9723096319323098  0.3526313384196837   T   T
T
  0.0655611575675096  0.2222410896475299  0.3514198547552678   T   T
T
  0.8162693778262649  0.9723326366491887  0.3526899214883804   T   T
T
  0.0607443733316306  0.7198059599564637  0.3537999600868440   T   T
T
  0.0650074284210752  0.4716398515778673  0.3526802117851227   T   T
T
  0.3155734148475176  0.9723828714843956  0.3522297315508310   T   T
T
  0.5657628286126716  0.9723499101903189  0.3522373831252586   T   T
T
  0.8161923074844922  0.2228496559525656  0.3526973741142978   T   T
T
  0.8036574894914325  0.4843264513889958  0.3510600531847675   T   T
T
  0.5681285272933899  0.2272636641942930  0.3538643052132622   T   T
T
  0.3155129605921751  0.4716334332601509  0.3527074420975857   T   T
T
  0.5896865780255222  0.4843762653404979  0.3510639401502402   T   T
T
  0.5680986278302040  0.7198174722249191  0.3538642468236469   T   T
T
```

```
  0.4079130317856582  0.3934900964154231  0.4315183823968028   T   T
T
  0.8844937608156475  0.6317437457067874  0.4364955935953743   T   T
T
  0.8945343221670434  0.8800378221246273  0.4314868083713055   T   T
T
  0.1480218583174194  0.8873793788911750  0.4349094589952149   T   T
T
  0.4004950368679037  0.1398936002498559  0.4349349893700628   T   T
T
  0.1483356858567497  0.1395496343474041  0.4350173659499092   T   T
T
  0.3989316372598365  0.8889327490050682  0.4357456576607972   T   T
T
  0.1481097038492371  0.6395881475082593  0.4349597707134721   T   T
T
  0.9001551078740264  0.1395709383463885  0.4350322800287846   T   T
T
  0.8944992290149854  0.3933963209191378  0.4315122500587129   T   T
T
  0.6482613880704532  0.1397861491836958  0.4349762325288343   T   T
T
  0.6482770517784897  0.8873945193770915  0.4349526386872303   T   T
T
  0.1482681373349643  0.3876690992412496  0.4350386271428892   T   T
T
  0.4005252534197465  0.6396324638162817  0.4349557021928210   T   T
T
  0.6562829416400134  0.4036024427684571  0.4365171327653730   T   T
T
  0.6564093894442926  0.6317740669578518  0.4365277466561885   T   T
T
  0.7323903988249435  0.5557005427204665  0.3973234565180996   T   T
T
```

Pt_2@CeO_2

```
System
   1.00000000000000
    13.4388805250000001   -7.7589412890000000    0.0000000000000000
     0.0000000000000000   15.5178825770000000    0.0000000000000000
     0.0000000000000000    0.0000000000000000   19.0058000000000007
   Ce    O    Pt
    46    96     2
Selective dynamics
Direct
  0.1488931311114570  0.1389316520428210  0.0621532900483004   F   F
F
  0.1488931311114570  0.3889316105262068  0.0621532900483004   F   F
F
  0.3988931213450186  0.1389316679259665  0.0621532900483004   F   F
F
  0.3988931213450186  0.3889316264093523  0.0621532900483004   F   F
F
  0.1488931311114570  0.6389316334513708  0.0621532900483004   F   F
F
  0.1488931311114570  0.8889316563765348  0.0621532900483004   F   F
F
```

0.3988931213450186 0.6389316493345163 0.0621532900483004 F F
F
0.3988931213450186 0.8889316078178950 0.0621532900483004 F F
F
0.6488931115785803 0.1389316838091119 0.0621532900483004 F F
F
0.6488931115785803 0.3889316422924978 0.0621532900483004 F F
F
0.8988931018121420 0.1389316996922574 0.0621532900483004 F F
F
0.8988931018121420 0.3889316581756432 0.0621532900483004 F F
F
0.6488931115785803 0.6389316007758765 0.0621532900483004 F F
F
0.6488931115785803 0.8889316237010405 0.0621532900483004 F F
F
0.8988931018121420 0.6389316166590220 0.0621532900483004 F F
F
0.8988931018121420 0.8889316395841860 0.0621532900483004 F F
F
0.3201100592792359 0.2249138358878793 0.2267221002984880 T T
T
0.8156303629763404 0.9713346549219444 0.2276464893870279 T T
T
0.0654142051003964 0.9724846522511085 0.2286640783177570 T T
T
0.8132910750438219 0.4730192224045963 0.2264529895209706 T T
T
0.0664978648338526 0.2222262443986211 0.2276529572569227 T T
T
0.8129718751162142 0.7177473100449246 0.2267166669302829 T T
T
0.0647903277246005 0.7220462710100328 0.2287060098662834 T T
T
0.3158557317607153 0.9731117566212768 0.2286936971739181 T T
T
0.8152760765240085 0.2225788246859022 0.2288302944646411 T T
T
0.0647608037733865 0.4718422729806961 0.2292365216052313 T T
T
0.3155847306870551 0.7222858184603038 0.2295126723364572 T T
T
0.5648548591633282 0.2245855999759818 0.2264529228535203 T T
T
0.5660306314908264 0.9730922236680647 0.2292296759658014 T T
T
0.3166648574876362 0.4721889606791959 0.2286729447237551 T T
T
0.5669515465362742 0.4709090571107666 0.2244854203536753 T T
T
0.5656682740588513 0.7211693648160971 0.2286676652595059 T T
T
0.9767095024696795 0.7997086147117431 0.3940293166275789 T T
T
0.2382727358700911 0.0612964947431656 0.3940243392514521 T T
T
0.2389015269418259 0.3079576434116405 0.3933125104640033 T T
T

0.2316035873788164	0.8063013023686094	0.3966870427026167	T	T	T
0.9820929807892180	0.0557841736797328	0.3913393568478007	T	T	T
0.7299552059842895	0.7989340790195649	0.3933051429251403	T	T	T
0.9757934522772151	0.5542522052420211	0.3952081097045632	T	T	T
0.4836405699616982	0.0620998366959864	0.3952063111630543	T	T	T
0.7328506324660632	0.0546977945336431	0.3928279662766300	T	T	T
0.2326821379081167	0.5562856751686681	0.3955670355497471	T	T	T
0.9831563281906260	0.3050325559401370	0.3928407687594199	T	T	T
0.4815930190515048	0.8051692141822976	0.3955568331621242	T	T	T
0.7268336193246780	0.3110690125602055	0.3939023307189778	T	T	T
0.4870845341793799	0.5508080178451442	0.3954295320581808	T	T	T
0.2320597308816481	0.0555286540517605	0.0209323332966886	T	T	T
0.2322264115820047	0.3055982715224701	0.0205499900030546	F	F	F
0.4822264762265220	0.0555983661277182	0.0205499900030546	F	F	F
0.4822264762265220	0.3055983246110969	0.0205499900030546	F	F	F
0.2322264115820047	0.5555982944476341	0.0205499900030546	F	F	F
0.2322264115820047	0.8055982529310199	0.0205499900030546	F	F	F
0.4822264762265220	0.5555982830944828	0.0205499900030546	F	F	F
0.4822264762265220	0.8055983060196468	0.0205499900030546	F	F	F
0.7322263920491281	0.0555982803635970	0.0205499900030546	F	F	F
0.7322263920491281	0.3055983032887610	0.0205499900030546	F	F	F
0.9822264566936454	0.0555983334522239	0.0205499900030546	F	F	F
0.9822264566936454	0.3055983563773879	0.0205499900030546	F	F	F
0.7322263920491281	0.5555982617721469	0.0205499900030546	F	F	F
0.7322263920491281	0.8055982846973109	0.0205499900030546	F	F	F
0.9822264566936454	0.5555983148607737	0.0205499900030546	F	F	F
0.9822264566936454	0.8055983377859306	0.0205499900030546	F	F	F
0.0655597762299465	0.9722648937330050	0.1037486977659441	F	F	F
0.0655597762299465	0.2222649538410764	0.1037486977659441	F	F	F

```
  0.3155597664635081  0.9722649740579286  0.1037486977659441   F   F
F
  0.3155597664635081  0.2222649697242218  0.1037486977659441   F   F
F
  0.0655597762299465  0.4722649767662404  0.1037486977659441   F   F
F
  0.0655597762299465  0.7222648708078410  0.1037486977659441   F   F
F
  0.3155597664635081  0.4722649926493858  0.1037486977659441   F   F
F
  0.3155597664635081  0.7222649511327646  0.1037486977659441   F   F
F
  0.5655597566970698  0.9722649254992959  0.1037486977659441   F   F
F
  0.5655597566970698  0.2222649211655821  0.1037486977659441   F   F
F
  0.8155597469306315  0.9722649413824414  0.1037486977659441   F   F
F
  0.8155597469306315  0.2222650014905128  0.1037486977659441   F   F
F
  0.5655597566970698  0.4722649440907460  0.1037486977659441   F   F
F
  0.5655597566970698  0.7222649025741319  0.1037486977659441   F   F
F
  0.8155597469306315  0.4722649599738915  0.1037486977659441   F   F
F
  0.8155597469306315  0.7222649184572774  0.1037486977659441   F   F
F
  0.1489850286469476  0.8889614302570976  0.1855009523408668   F   F
F
  0.1479368211851665  0.1383443638469752  0.1854432201320165   T   T
T
  0.8995029433450249  0.8899039759061346  0.1854406233279781   T   T
T
  0.9056131352911989  0.6421605951355928  0.1846569241159290   T   T
T
  0.8990506416199843  0.1387805463916997  0.1861133997811473   T   T
T
  0.8993504628710310  0.3882615453436522  0.1859250340295898   T   T
T
  0.6496038385542482  0.1384937482599650  0.1859063888393246   T   T
T
  0.3950039602854308  0.3919461978326392  0.1836073466080489   T   T
T
  0.1494925932602967  0.3887474872945012  0.1867331325314365   T   T
T
  0.1483505952418523  0.6389448103982523  0.1876692482208568   T   T
T
  0.3957366804825972  0.1322720325727240  0.1846322761679562   T   T
T
  0.6491040629085991  0.8883367581530288  0.1867300228705783   T   T
T
  0.3993581233726908  0.6385029411674039  0.1872618488196844   T   T
T
  0.6528653472700783  0.3849933970169505  0.1818873510735086   T   T
T
  0.3989250804032846  0.8895058929419821  0.1876579548396525   T   T
T
```

0.6459049063951376	0.6428380624494997	0.1836102976547677	T	T	T
0.2324994539276136	0.0587980975748188	0.2732550158535124	T	T	T
0.9822499051047194	0.0555871695338486	0.2707111445037133	T	T	T
0.2308464686929678	0.8071011105017125	0.2734951375360148	T	T	T
0.7306046025262211	0.5545956452624770	0.2463332706607484	T	T	T
0.2332827247343321	0.3033135701452079	0.2728886868422188	T	T	T
0.9791416704215212	0.8054339136803497	0.2732476543888088	T	T	T
0.7345561464315412	0.8045713048897105	0.2728774324014481	T	T	T
0.4853023285857213	0.5525551057218427	0.2737543845359625	T	T	T
0.7323776562084738	0.0550707312219066	0.2712148868292099	T	T	T
0.9827704008467946	0.3054774078548599	0.2712052709220754	T	T	T
0.2327150123141012	0.5549135770336371	0.2725484832910512	T	T	T
0.7326037174545555	0.3052356986475900	0.2736578933875342	T	T	T
0.9777818500861730	0.5523068937669660	0.2738550899752171	T	T	T
0.4832702971876815	0.3072613227845771	0.2463478437173283	T	T	T
0.4855969996996544	0.0600987515525943	0.2738632913609246	T	T	T
0.4829531751727250	0.8051495440411219	0.2725514782096890	T	T	T
0.3442139455628447	0.2382902998029683	0.3512874618548931	T	T	T
0.7996947426736412	0.6937015045874906	0.3512930490074889	T	T	T
0.3150639822559492	0.7227836819657104	0.3523861999803826	T	T	T
0.0645490726696454	0.9733801681600682	0.3539521021496445	T	T	T
0.0657139098903506	0.2212298039622762	0.3521597930608334	T	T	T
0.8166501635450796	0.9721318175961443	0.3521558704684376	T	T	T
0.0584808193064715	0.7187425670640667	0.3549596554859897	T	T	T
0.0640163087589390	0.4708678176120993	0.3535189229684308	T	T	T
0.3191643408633212	0.9794534780975180	0.3549564786636997	T	T	T
0.5670108955079938	0.9738298028988308	0.3535432271578270	T	T	T
0.8165978272189569	0.2213010224432580	0.3536838384872775	T	T	T
0.7956612595286293	0.4770703621849800	0.3528248130794356	T	T	T

```
  0.5608563582863529  0.2422904489743725  0.3528426828089885   T   T
T
  0.3191728654647648  0.4703429866117656  0.3548679513910225   T   T
T
  0.5765726511746400  0.4613843299337603  0.3519852443107958   T   T
T
  0.5675336293198571  0.7186677637017512  0.3548476009267920   T   T
T
  0.4159048116388262  0.3890433983227525  0.4340808492449039   T   T
T
  0.8781813048527940  0.6264864834497269  0.4362948930810495   T   T
T
  0.8921000497513446  0.8770210546191904  0.4306657741243703   T   T
T
  0.1495395428207459  0.8883606091943904  0.4343081558704446   T   T
T
  0.4113928027490117  0.1597370160818077  0.4362880133579434   T   T
T
  0.1609058302229950  0.1458819286319527  0.4306677172242522   T   T
T
  0.3981767905497198  0.8901200500153329  0.4355733605328403   T   T
T
  0.1477850615645985  0.6396987794877239  0.4355882497998548   T   T
T
  0.9005210374107847  0.1373121622104519  0.4351239879869537   T   T
T
  0.8919317979678192  0.3937068629809610  0.4314562085650931   T   T
T
  0.6441769426886267  0.1459793485290518  0.4314431287693253   T   T
T
  0.6495688916407537  0.8876277755366739  0.4344569430755861   T   T
T
  0.1502203760743841  0.3883054997721398  0.4344758682181724   T   T
T
  0.3980344224681052  0.6398584821451824  0.4354208357710768   T   T
T
  0.6408250411283094  0.3971286608458267  0.4418735759678797   T   T
T
  0.6489571263763397  0.6220834523920514  0.4340691055779219   T   T
T
  0.7238395567945810  0.5471593892768962  0.3979648666050764   T   T
T
  0.4908138494896566  0.3141670710795426  0.3979741644308749   T   T
T
```

Pt_1@CeO_2 CO ads

```
System
   1.00000000000000
    13.4388805250000001   -7.7589412890000000    0.0000000000000000
     0.0000000000000000   15.5178825770000000    0.0000000000000000
     0.0000000000000000    0.0000000000000000   19.0058000000000007
   Ce    O    Pt    C
    47    97     1     1
Selective dynamics
Direct
```

```
  0.1488931311114570  0.1389316520428210  0.0621532900483004   F   F
F
  0.1488931311114570  0.3889316105262068  0.0621532900483004   F   F
F
  0.3988931213450186  0.1389316679259665  0.0621532900483004   F   F
F
  0.3988931213450186  0.3889316264093523  0.0621532900483004   F   F
F
  0.1488931311114570  0.6389315045678075  0.0621532900483004   F   F
F
  0.1488931311114570  0.8889315274929714  0.0621532900483004   F   F
F
  0.3988931213450186  0.6389315204509529  0.0621532900483004   F   F
F
  0.3988931213450186  0.8889316078178950  0.0621532900483004   F   F
F
  0.6488931115785803  0.1389316838091119  0.0621532900483004   F   F
F
  0.6488931115785803  0.3889315134089344  0.0621532900483004   F   F
F
  0.8988931018121420  0.1389316996922574  0.0621532900483004   F   F
F
  0.8988931018121420  0.3889316581756432  0.0621532900483004   F   F
F
  0.6488931115785803  0.6389314718923131  0.0621532900483004   F   F
F
  0.6488931115785803  0.8889316237010405  0.0621532900483004   F   F
F
  0.8988931018121420  0.6389316166590220  0.0621532900483004   F   F
F
  0.8988931018121420  0.8889316395841860  0.0621532900483004   F   F
F
  0.3153351351785694  0.2211228731632063  0.2287495980275264   T   T
T
  0.8169117583321374  0.9740706440872645  0.2274438784141297   T   T
T
  0.0664049314587292  0.9721982327835539  0.2287822049393011   T   T
T
  0.8146009108048005  0.4730194385590748  0.2304346235550627   T   T
T
  0.0655180293409113  0.2221977671860188  0.2279709547804039   T   T
T
  0.8191299994046821  0.7252360449930118  0.2274208162501399   T   T
T
  0.0657676600598312  0.7227380161525899  0.2277999892982177   T   T
T
  0.3161809888899276  0.9714205117031054  0.2273792819624624   T   T
T
  0.8151720097261052  0.2237431667371641  0.2288639715162449   T   T
T
  0.0639159979515213  0.4725633828121626  0.2288642278924325   T   T
T
  0.3158469610482726  0.7219146050782089  0.2275420255833311   T   T
T
  0.5648929598371171  0.2217352054761131  0.2277432259601718   T   T
T
  0.5658295746131403  0.9718417774846021  0.2275587629865767   T   T
T
```

0.3135496944053281	0.4708081020062614	0.2273863669551177	T	T	T
0.5623090886086455	0.4685109389678079	0.2273643979998140	T	T	T
0.5662074047366724	0.7214751297251085	0.2280459561215725	T	T	T
0.9916082390600194	0.8045969254751765	0.3993516969540180	T	T	T
0.2324399810820194	0.0546511861895425	0.3942510309190588	T	T	T
0.2304157568096927	0.3039932343652483	0.3943332913407936	T	T	T
0.2355681760594958	0.8055973699957215	0.3934495665333843	T	T	T
0.9834280133529104	0.0568388532706496	0.3943708271804973	T	T	T
0.7356929534531675	0.8130781043483590	0.3937053147153777	T	T	T
0.9849259544600016	0.5617933529037289	0.3990176497567556	T	T	T
0.4820721282089437	0.0519987805671667	0.3934431522852891	T	T	T
0.7332359862758336	0.0573882141441020	0.3940179744559980	T	T	T
0.2303101423354611	0.5544796834373145	0.3939938130157227	T	T	T
0.9807804245332215	0.3067587587190159	0.3938777783306400	T	T	T
0.4828827654821705	0.8048588575896670	0.3930683862716679	T	T	T
0.7256756338038222	0.3027330615644520	0.3988070768666186	T	T	T
0.4742955964635445	0.5517491117535016	0.3935298216832559	T	T	T
0.4822828093628533	0.2955835060734525	0.3990003136617800	T	T	T
0.2322408650934831	0.0555979208510089	0.0208891511067194	T	T	T
0.2319847991951676	0.3057102290442089	0.0209568131833464	F	F	F
0.4822265506374848	0.0555984033331924	0.0205499900030546	F	F	F
0.4822265506374848	0.3055983618165783	0.0205499900030546	F	F	F
0.2322264115820047	0.5555982944476341	0.0205499900030546	F	F	F
0.2322264115820047	0.8055981884892347	0.0205499900030546	F	F	F
0.4822265506374848	0.5555982558581789	0.0205499900030546	F	F	F
0.4822265506374848	0.8055982787833429	0.0205499900030546	F	F	F
0.7322263920491281	0.0555984092471604	0.0205499900030546	F	F	F
0.7322263920491281	0.3055983032887610	0.0205499900030546	F	F	F
0.9822264566936454	0.0555984623357872	0.0205499900030546	F	F	F

```
  0.9822264566936454  0.3055983563773879  0.0205499900030546   F   F
F
  0.7322263920491281  0.5555983262139250  0.0205499900030546   F   F
F
  0.7322263920491281  0.8055982846973109  0.0205499900030546   F   F
F
  0.9822264566936454  0.5555983148607737  0.0205499900030546   F   F
F
  0.9822264566936454  0.8055983377859306  0.0205499900030546   F   F
F
  0.0655597762299465  0.9722647648494416  0.1037486977659441   F   F
F
  0.0655597762299465  0.2222649538410764  0.1037486977659441   F   F
F
  0.3155597664635081  0.9722648451743652  0.1037486977659441   F   F
F
  0.3155597664635081  0.2222649697242218  0.1037486977659441   F   F
F
  0.0655597762299465  0.4722649767662404  0.1037486977659441   F   F
F
  0.0655597762299465  0.7222648708078410  0.1037486977659441   F   F
F
  0.3155597664635081  0.4722649926493858  0.1037486977659441   F   F
F
  0.3155597664635081  0.7222648222492012  0.1037486977659441   F   F
F
  0.5655597566970698  0.9722649254992959  0.1037486977659441   F   F
F
  0.5655597566970698  0.2222649211655821  0.1037486977659441   F   F
F
  0.8155597469306315  0.9722649413824414  0.1037486977659441   F   F
F
  0.8155597469306315  0.2222650014905128  0.1037486977659441   F   F
F
  0.5655597566970698  0.4722649440907460  0.1037486977659441   F   F
F
  0.5655597566970698  0.7222647736905685  0.1037486977659441   F   F
F
  0.8155597469306315  0.4722649599738915  0.1037486977659441   F   F
F
  0.8155597469306315  0.7222649184572774  0.1037486977659441   F   F
F
  0.1489850286469476  0.8889614302570976  0.1855009523408668   F   F
F
  0.1488304859919759  0.1388807209196322  0.1856984658072494   T   T
T
  0.8989014160698283  0.8899788024660893  0.1850532274175585   T   T
T
  0.8967416980215628  0.6379706282784119  0.1866031836677983   T   T
T
  0.8992725634339898  0.1392282460008059  0.1858351470349554   T   T
T
  0.8980500318820915  0.3896338203843802  0.1859727798101436   T   T
T
  0.6497175895670358  0.1407747583797022  0.1859122143768288   T   T
T
  0.3977194796017733  0.3887136653502321  0.1849764515388576   T   T
T
```

0.1484182861413954	0.3884684549814544	0.1858306461055330	T	T	T
0.1468722898938984	0.6379932905782716	0.1859038501212549	T	T	T
0.3991733428259586	0.1383511960064332	0.1852755574307004	T	T	T
0.6506091175747289	0.8874964236901198	0.1865020677702862	T	T	T
0.4002621154064558	0.6371873283289976	0.1864423468963189	T	T	T
0.6495987061259247	0.3908584199248104	0.1865317283592096	T	T	T
0.3993264229428250	0.8883231716455586	0.1841024478480044	T	T	T
0.6473887019446944	0.6402145000975547	0.1841511411466318	T	T	T
0.2327617131942355	0.0548710835899329	0.2704505081545232	T	T	T
0.9828843001394362	0.0554340676350033	0.2710065359838177	T	T	T
0.2351365081826331	0.8055339784990458	0.2689227983154293	T	T	T
0.7242025460495453	0.5634605730118749	0.2704348899526447	T	T	T
0.2321854926269471	0.3047882185007737	0.2709286787563672	T	T	T
0.9834100024413202	0.8082018745878337	0.2695411443070094	T	T	T
0.7373043100453980	0.8041244214198923	0.2741027653857728	T	T	T
0.4833048872042344	0.5503374024312544	0.2739721860839714	T	T	T
0.7323079923781187	0.0564174330257665	0.2696773222019981	T	T	T
0.9814371338829292	0.3062196134811147	0.2708070146723658	T	T	T
0.2312488781841726	0.5554316837743226	0.2696745448318578	T	T	T
0.7346927164031823	0.3107075664550045	0.2732173685065510	T	T	T
0.9769387428947032	0.5529968578469702	0.2732942079757819	T	T	T
0.4789971706142278	0.3037979370700719	0.2695319233868589	T	T	T
0.4820354763889139	0.0522572873515027	0.2690009741624564	T	T	T
0.4819382317431737	0.8058029729081699	0.2685641897992737	T	T	T
0.3138994503499071	0.2192335795885275	0.3539672971180448	T	T	T
0.8329099353425612	0.7069780455095320	0.3583991494739356	T	T	T
0.3177059811219833	0.7215797761079624	0.3515550834984821	T	T	T
0.0679944049351610	0.9731294969467356	0.3539884294522593	T	T	T
0.0648649077139535	0.2226323835808618	0.3514595631899376	T	T	T

0.8159058089361091 0.9749633786075960 0.3507386506694519 T T
T
0.0725869997316606 0.7265090205809813 0.3478666621883814 T T
T
0.0662772949674657 0.4728537948626111 0.3526405936983968 T T
T
0.3167085793813283 0.9706760068836613 0.3509171759362703 T T
T
0.5662596217254220 0.9700011044483755 0.3515774270135983 T T
T
0.8147207499551923 0.2212924281793021 0.3525802284647920 T T
T
0.7992232108449103 0.4877947116509227 0.3559995357281671 T T
T
0.5610452439233513 0.2148445152946527 0.3477443428278082 T T
T
0.3124975327426601 0.4716372175019631 0.3506549611629426 T T
T
0.5796461779194144 0.4546286478902450 0.3578748486371437 T T
T
0.5623924814186161 0.7251510372846058 0.3505818962786582 T T
T
0.3987419053146055 0.3924366828379813 0.4329203615661131 T T
T
0.9486118097867388 0.6609706106656787 0.4534836846312773 T T
T
0.8947773993970135 0.8884511532370537 0.4329915272724990 T T
T
0.1552209862188481 0.8909210069146624 0.4371960246265211 T T
T
0.3963416887298172 0.1320993793163716 0.4369822009812674 T T
T
0.1471051720677177 0.1400282839246462 0.4366052566607971 T T
T
0.4003261398910907 0.8873819163383647 0.4353736671341384 T T
T
0.1532515962185056 0.6404864745682843 0.4353319218374800 T T
T
0.8993896031859683 0.1400194748330341 0.4356435750085024 T T
T
0.8951537599038636 0.3923776447030127 0.4317829100393993 T T
T
0.6470936078329740 0.1344108263701352 0.4353356503050708 T T
T
0.6521750685486876 0.8884478538259869 0.4361510347370664 T T
T
0.1475099598603253 0.3881280167136848 0.4357006317349333 T T
T
0.3989881747815909 0.6352853106635797 0.4361328496164703 T T
T
0.6262217784515040 0.3391092381568728 0.4530443432469463 T T
T
0.6487854607946648 0.6386101253208409 0.4085963675353398 T T
T
0.7330233670245352 0.5484921877308621 0.5352289527362667 T T
T
0.7225802225512694 0.5642712259214534 0.3774757323703849 T T
T

```
  0.7303803925722376  0.5536266433529148  0.4749342979858506   T   T
T
```

Pt_2@CeO_2 CO ads

```
System
   1.00000000000000
    13.4388805250000001   -7.7589412890000000    0.0000000000000000
     0.0000000000000000   15.5178825770000000    0.0000000000000000
     0.0000000000000000    0.0000000000000000   19.0058000000000007
   Ce   O    Pt   C
    46    97     2     1
Selective dynamics
Direct
  0.1488931311114570  0.1389316520428210  0.0621532900483004   F   F
F
  0.1488931311114570  0.3889316105262068  0.0621532900483004   F   F
F
  0.3988931213450186  0.1389316679259665  0.0621532900483004   F   F
F
  0.3988931213450186  0.3889316264093523  0.0621532900483004   F   F
F
  0.1488931311114570  0.6389315690095856  0.0621532900483004   F   F
F
  0.1488931311114570  0.8889315919347496  0.0621532900483004   F   F
F
  0.3988931213450186  0.6389315848927311  0.0621532900483004   F   F
F
  0.3988931213450186  0.8889316078178950  0.0621532900483004   F   F
F
  0.6488931115785803  0.1389316838091119  0.0621532900483004   F   F
F
  0.6488931115785803  0.3889315778507125  0.0621532900483004   F   F
F
  0.8988931018121420  0.1389316996922574  0.0621532900483004   F   F
F
  0.8988931018121420  0.3889316581756432  0.0621532900483004   F   F
F
  0.6488931115785803  0.6389315363340984  0.0621532900483004   F   F
F
  0.6488931115785803  0.8889316237010405  0.0621532900483004   F   F
F
  0.8988931018121420  0.6389316166590220  0.0621532900483004   F   F
F
  0.8988931018121420  0.8889316395841860  0.0621532900483004   F   F
F
  0.3173566589856404  0.2226033076541669  0.2267260212504200   T   T
T
  0.8165864162693683  0.9733523366085894  0.2270972333471568   T   T
T
  0.0662863987136930  0.9722408620576544  0.2290810770744563   T   T
T
  0.8144248290719539  0.4725653379000635  0.2297378927534562   T   T
T
  0.0659116153079472  0.2221578112686924  0.2282713793912028   T   T
T
```

0.8174166151095268	0.7229630395286766	0.2283496318347386	T	T	T
0.0659671751920751	0.7227399417548837	0.2282529322158641	T	T	T
0.3167837004610514	0.9725066343994101	0.2275596646703509	T	T	T
0.8147744244438053	0.2233928647129807	0.2292842826511854	T	T	T
0.0643740822750810	0.4725884267660250	0.2295822305964463	T	T	T
0.3166184292021167	0.7225796545251099	0.2285109906507918	T	T	T
0.5641416924337250	0.2239903780002740	0.2261096299490248	T	T	T
0.5663479050724298	0.9732208519281166	0.2283195337967820	T	T	T
0.3140817074129594	0.4711194645466245	0.2276555261842393	T	T	T
0.5615344121214788	0.4678486228483346	0.2253049404531743	T	T	T
0.5661318191750476	0.7213347928477865	0.2287875810034569	T	T	T
0.9904842616280760	0.8035807187847647	0.3986482146466342	T	T	T
0.2377715041207726	0.0600794204483773	0.3936834038428189	T	T	T
0.2354840524210476	0.3039068002905584	0.3940648047632682	T	T	T
0.2365870707614353	0.8072003501154260	0.3945279496067589	T	T	T
0.9823564358771903	0.0557706206770385	0.3926622441894451	T	T	T
0.7340346376475317	0.8106963116303815	0.3934321681097260	T	T	T
0.9867175840153816	0.5619813636188897	0.3996317246402893	T	T	T
0.4821081673943802	0.0581586999763849	0.3941153738515595	T	T	T
0.7335390685777139	0.0582409207822707	0.3930294001513255	T	T	T
0.2322319737092199	0.5542153606211866	0.3954099306764371	T	T	T
0.9810935910096806	0.3065816478335779	0.3937417139089348	T	T	T
0.4836951015276045	0.8059665595054257	0.3935728349021715	T	T	T
0.7228284790835222	0.3094454195751705	0.3976753585693923	T	T	T
0.4756616428770041	0.5464177875219495	0.3942225240487525	T	T	T
0.2320550143271713	0.0554979457157823	0.0209304475718048	T	T	T
0.2319848232162633	0.3057102544592537	0.0209568131396391	T	T	T
0.4822265506374848	0.0555984033331924	0.0205499900030546	F	F	F
0.4822265506374848	0.3055983618165783	0.0205499900030546	F	F	F

0.2322264115820047 0.5555982944476341 0.0205499900030546 F F
F
0.2322264115820047 0.8055981884892347 0.0205499900030546 F F
F
0.4822265506374848 0.5555983202999570 0.0205499900030546 F F
F
0.4822265506374848 0.8055983432251210 0.0205499900030546 F F
F
0.7322263920491281 0.0555983448053823 0.0205499900030546 F F
F
0.7322263920491281 0.3055983032887610 0.0205499900030546 F F
F
0.9822264566936454 0.0555983978940091 0.0205499900030546 F F
F
0.9822264566936454 0.3055983563773879 0.0205499900030546 F F
F
0.7322263920491281 0.5555983262139250 0.0205499900030546 F F
F
0.7322263920491281 0.8055982846973109 0.0205499900030546 F F
F
0.9822264566936454 0.5555983148607737 0.0205499900030546 F F
F
0.9822264566936454 0.8055983377859306 0.0205499900030546 F F
F
0.0655597762299465 0.9722648292912197 0.1037486977659441 F F
F
0.0655597762299465 0.2222649538410764 0.1037486977659441 F F
F
0.3155597664635081 0.9722649096161504 0.1037486977659441 F F
F
0.3155597664635081 0.2222649697242218 0.1037486977659441 F F
F
0.0655597762299465 0.4722649767662404 0.1037486977659441 F F
F
0.0655597762299465 0.7222648708078410 0.1037486977659441 F F
F
0.3155597664635081 0.4722649926493858 0.1037486977659441 F F
F
0.3155597664635081 0.7222648866909864 0.1037486977659441 F F
F
0.5655597566970698 0.9722649254992959 0.1037486977659441 F F
F
0.5655597566970698 0.2222649211655821 0.1037486977659441 F F
F
0.8155597469306315 0.9722649413824414 0.1037486977659441 F F
F
0.8155597469306315 0.2222650014905128 0.1037486977659441 F F
F
0.5655597566970698 0.4722649440907460 0.1037486977659441 F F
F
0.5655597566970698 0.7222648381323467 0.1037486977659441 F F
F
0.8155597469306315 0.4722649599738915 0.1037486977659441 F F
F
0.8155597469306315 0.7222649184572774 0.1037486977659441 F F
F
0.1489850286469476 0.8889614302570976 0.1855009523408668 F F
F

0.1479368014546765	0.1383443228363603	0.1854432331183133	F	F	F
0.8986580697801592	0.8892322340092618	0.1854792220882875	T	T	T
0.8965817968330430	0.6377221029224028	0.1870738090599140	T	T	T
0.8990044737174824	0.1391025342958450	0.1861085151393773	T	T	T
0.8973440846440258	0.3890406753752190	0.1868206921304543	T	T	T
0.6503159416451313	0.1404710678642791	0.1853877020823546	T	T	T
0.3944648542434060	0.3920983534078488	0.1837337544535753	T	T	T
0.1487676450586746	0.3887890084261824	0.1863924216803731	T	T	T
0.1476562946394315	0.6382869069608577	0.1869858540285824	T	T	T
0.3962514784795599	0.1322022321930206	0.1839671365363620	T	T	T
0.6505966139016615	0.8877651141159493	0.1869119340970160	T	T	T
0.4002295721930542	0.6378157961603282	0.1869230002150461	T	T	T
0.6562983072922034	0.3938990045297308	0.1844412479299758	T	T	T
0.4003249951019207	0.8900514626622898	0.1853085207641439	T	T	T
0.6483492712038017	0.6412269558188010	0.1840888269869140	T	T	T
0.2326935337225695	0.0570718167451693	0.2717545387939477	T	T	T
0.9822830765914972	0.0550921124763351	0.2708804267291667	T	T	T
0.2366675849671470	0.8076799137924601	0.2701336629669478	T	T	T
0.7214347071798091	0.5595620796682699	0.2714757915397240	T	T	T
0.2314437495644293	0.3025586861842039	0.2720653335041822	T	T	T
0.9820126741703485	0.8069125706493211	0.2710547814160287	T	T	T
0.7371910631761992	0.8035860538925487	0.2744055877920351	T	T	T
0.4842541036444365	0.5517174498674142	0.2745566452221013	T	T	T
0.7324314557501395	0.0567186864602344	0.2701946884787801	T	T	T
0.9819993474030712	0.3063607828004835	0.2711723770133240	T	T	T
0.2326663346507035	0.5551082745558475	0.2715608732278638	T	T	T
0.7303803864078767	0.3073518463241537	0.2752629968372851	T	T	T
0.9768978389098361	0.5528268199777300	0.2738652381547146	T	T	T
0.4816296715770582	0.3055931241685772	0.2440741789545087	T	T	T

0.4863213970427689	0.0579023623233435	0.2717886404745052	T	T	T
0.4825871837001849	0.8066200926149458	0.2695544215534555	T	T	T
0.3371341637103608	0.2330516844353482	0.3498258320571505	T	T	T
0.8258976068469435	0.7016594445422064	0.3600539511698347	T	T	T
0.3188251606520186	0.7225877177975171	0.3523649130699772	T	T	T
0.0674986746453852	0.9741808296332649	0.3541513389264366	T	T	T
0.0643757471650653	0.2212503027956974	0.3523468354177558	T	T	T
0.8153705268677144	0.9743841817970397	0.3504646133786078	T	T	T
0.0733458701000170	0.7263623242730149	0.3478481697634268	T	T	T
0.0663870739886400	0.4723940938211405	0.3533433947678959	T	T	T
0.3200654156753798	0.9765647529435805	0.3526344018564245	T	T	T
0.5671963250871207	0.9718686026095709	0.3528369205315319	T	T	T
0.8155259017234827	0.2220871881234605	0.3529988580819526	T	T	T
0.7954686877839463	0.4821085144895782	0.3568540034097064	T	T	T
0.5479634719228555	0.2281541809565332	0.3495342650307064	T	T	T
0.3155253325483531	0.4678079446080232	0.3530561648038972	T	T	T
0.5631640052721046	0.4356592920834726	0.3543700050524277	T	T	T
0.5648281958458731	0.7268294145958978	0.3517201211926571	T	T	T
0.4074111875069371	0.3831622176643300	0.4357198264195921	T	T	T
0.9490170656044943	0.6614492827624540	0.4527914383375704	T	T	T
0.8936764311687964	0.8871145899973447	0.4318110723317529	T	T	T
0.1564025114064057	0.8921885286756724	0.4355352961048805	T	T	T
0.4006092952379779	0.1484155108037450	0.4371135378303843	T	T	T
0.1567277793409802	0.1440615367844446	0.4322638561221316	T	T	T
0.4003318676181081	0.8900800421139647	0.4354669577773628	T	T	T
0.1542144440613924	0.6405247788379564	0.4358879616813265	T	T	T
0.8983272845035597	0.1385557506262379	0.4354028363921263	T	T	T
0.8928644700656807	0.3937638676857335	0.4317716888057988	T	T	T
0.6437158798593178	0.1425522237901495	0.4306214427325185	T	T	T

```
  0.6530146641939785  0.8897921255728062  0.4364530776246363   T   T
T
  0.1492183877523923  0.3883538078071211  0.4352357742256022   T   T
T
  0.3988041630253795  0.6331410552722401  0.4355183234412171   T   T
T
  0.6119205597616381  0.3421436002341441  0.4509526430787810   T   T
T
  0.6433175454682397  0.6298239743835178  0.4073206686010734   T   T
T
  0.7236969219089381  0.5361424173574559  0.5364415830357167   T   T
T
  0.7155635340576720  0.5535982180953637  0.3786170879416675   T   T
T
  0.4818595717041942  0.2974480082967613  0.4001989523512701   T   T
T
  0.7183910870512598  0.5376168272043568  0.4760555505601153   T   T
T
```

9. References

(1) Jayakumar, G.; Irudayaraj, A. A.; Raj, A. D. Particle Size Effect on the Properties of Cerium Oxide (CeO2) Manoparticles Synthesized by Hydrothermal Method. *Mech. Mater. Sci. Eng. J.* **2017**, 9, 2–7. https://doi.org/10.2412/mmse.3.4.481.

(2) ThermoFischer Scientific. *Iodine X-ray photoelectron spectra, iodine electron configuration, and other elemental information*.

(3) ThermoFischer Scientific. *Chlorine X-ray photoelectron spectra, chlorine electron configuration, and other elemental information*.

(4) Rakhmatullin, R. M.; Semashko, V. V.; Korableva, S. L.; Kiiamov, A. G.; Rodionov, A. A.; Tschaggelar, R.; van Bokhoven, J. A.; Paun, C. EPR Study of Ceria Nanoparticles Containing Different Concentration of Ce3+ Ions. *Mater. Chem. Phys.* **2018**, *219*, 251–257. https://doi.org/10.1016/j.matchemphys.2018.08.028.
